# A data-driven framework reconstructs the molecular continuum of human MASLD progression

**DOI:** 10.1038/s42255-026-01543-7

**Published:** 2026-07-14

**Authors:** Ioannis Kamzolas, Thodoris Koutsandreas, Charlie George Barker, Anna Vathrakokoili Pournara, Harry Weston, Naoto Fujiwara, Yujin Hoshida, Quentin M. Anstee, Michele Vacca, Irene Papatheodorou, Antonio Vidal-Puig, Evangelia Petsalaki

**Affiliations:** 1 European Molecular Biology Laboratory - European Bioinformatics Institute, Wellcome Genome Campus, Hinxton, UK; 2 MRC Institute of Metabolic Science, Metabolic Research Laboratories, University of Cambridge, Cambridge, UK; 3 Open Targets, Wellcome Genome Campus, Hinxton, UK; 4 Translational Genomics, Centre for Genomics Research, Discovery Sciences, BioPharmaceuticals R&D, AstraZeneca, Cambridge, UK; 5 Department of Gastroenterology and Hepatology, Mie University, Tsu city, Japan; 6 Division of Digestive and Liver Diseases, Department of Internal Medicine, Simmons Comprehensive Cancer Center, University of Texas Southwestern Medical Center, Dallas, TX USA; 7 Translational & Clinical Research Institute, Faculty of Medical Sciences, Newcastle University, Newcastle upon Tyne, UK; 8 Newcastle NIHR Biomedical Research Centre, Newcastle upon Tyne Hospitals NHS Trust, Newcastle upon Tyne, UK; 9 Clinica Medica “Cesare Frugoni”, Interdisciplinary Department of Medicine, University of Bari ”Aldo Moro”, Bari, Italy; 10 Roger Williams Institute of Liver Studies, School of Immunology & Microbial Sciences, Faculty of Life Sciences and Medicine, King’s College London, Foundation for Liver Research and King’s College Hospital, London, UK; 11 National Institute for Biostructure and Biosystems (INBB), Rome, Italy; 12 Centro de Investigacion Principe Felipe, Valencia, Spain; 13 Present Address: University College London Cancer Institute, London, UK; 14 Present Address: Wellcome Sanger Institute, Wellcome Genome Campus, Hinxton, Cambridge, UK; 15 Present Address: Medical School, University of East Anglia, Norwich, UK; 16 Present Address: Earlham Institute, Norwich, UK

**Keywords:** Systems analysis, Regulatory networks, Cellular signalling networks, Data integration, Metabolism

## Abstract

Metabolic dysfunction-associated steatotic liver disease (MASLD) progresses along a continuum from simple steatosis to steatohepatitis, fibrosis, cirrhosis and hepatocellular carcinoma. However, current clinical and research frameworks rely primarily on static, histology-defined stages that fail to capture the continuous nature of disease progression. Here, we present a data-driven framework that reconstructs MASLD progression as a continuous molecular trajectory from cross-sectional liver transcriptomic profiles. By positioning patients along this trajectory, we move beyond conventional stage-based classifications and resolve the ordered activation of regulatory programmes, signalling pathways and cellular remodelling processes underlying disease progression. To enable non-invasive patient stratification, we integrate the inferred molecular trajectory with paired liver-plasma proteomics data and identify a 57-gene plasma-accessible biomarker panel that accurately predicts advanced fibrosis and continuously positions patients along the disease trajectory across independent cohorts, outperforming established non-invasive clinical scores. Together, this work establishes a generalizable trajectory-based framework for understanding MASLD pathophysiology and provides a foundation for mechanistically informed biomarker discovery, precision staging and stage-aware therapeutic prioritization.

## Abstract (web summary)

A trajectory-based molecular framework reveals coordinated temporal shifts in metabolic, inflammatory and fibrogenic mechanisms across the human MASLD spectrum.

## Main

Metabolic dysfunction-Associated steatotic liver disease (MASLD) encompasses a spectrum of conditions, ranging from simple steatosis to steatohepatitis with progressive fibrosis (metabolic dysfunction-associated steatohepatitis; MASH) that occurs in the absence of alcohol excess and can progress to cirrhosis and hepatocellular carcinoma^1^. This global epidemic affects nearly one-third of western populations and is primarily driven by the rising prevalence of metabolic syndrome, type 2 diabetes and obesity^2^. Despite its prevalence, most MASLD cases remain asymptomatic and undiagnosed until late in the disease course.

Although histological assessment is considered the reference standard to diagnose and grade/stage MASLD using validated semi-quantitative scoring systems such as the non-alcoholic fatty liver disease (NAFLD) activity score developed by the NASH Clinical Research Network (NASH-CRN)^3^, its widespread adoption is limited due to perceived procedural risk, resource demands and issues related to both sampling and inter-observer variability^4^. In practice, diagnosis of MASLD relies on presence of cardio-metabolic risk factors coupled with imaging evidence of increased hepatic fat content (ultrasound and MRI-PDFF, i.e. protein density fat fraction) and evidence of hepatic fibrosis based on indirect biomarkers (for example, Fibrosis 4 (FIB-4)), direct extracellular matrix biomarkers (for example, Enhanced Liver Fibrosis test (ELF)) and hepatic elastography (for example, vibration controlled transient elastography by Fibroscan or MR elastography)^5^. Current American Association for the Study of Liver Diseases (AASLD) and European Association for the Study of the Liver, European Association for the Study of Diabetes and European Association for the Study of Obesity guidelines endorse non-invasive tests but emphasize the need for further improvements in non-invasive diagnostic strategies^6,7^. Consequently, there remains a need to develop more robust non-invasive biomarkers for MASLD.

Advances in systems biology and multi-omics have accelerated MASLD/MASH research for example^8,9^, with transcriptomic, proteomic and metabolomic studies linking the disease to metabolic syndrome, insulin resistance, and processes such as lipid accumulation, endoplasmic reticulum stress and oxidative stress^10^. Genome-wide association studies (GWAS) have identified major genetic risk factors, most notably the *PNPLA3* I148M variant^11^, which was later validated across large multi-ethnic cohorts^12^. Additional variants in *TM6SF2* (ref. ^13^), *MBOAT7* (ref.^14^) and *HSD17B13* (ref. ^15^) further define the genetic landscape, implicating lipid metabolism, inflammation and fibrosis pathways. Despite these breakthroughs, the complexity of MASLD progression, shaped by genetic, metabolic and environmental interactions, demands further research to elucidate stage-specific mechanisms and distinguish benign cases from progressive disease.

Recent studies have begun applying transcriptomic and systems-level analyses to stratify patients with MASLD and identify molecular signatures associated with disease severity and progression^16–18^. These efforts highlight the potential of molecular profiling to complement histological staging and uncover regulatory pathways involved in disease progression. However, most existing approaches rely on discrete comparisons between predefined disease stages or focus on predictive biomarker discovery, limiting their ability to capture the continuous molecular transitions underlying MASLD progression or to integrate regulatory networks with patient stratification.

Histology has long been a cornerstone for understanding MASLD pathophysiology, but its limitations as a sole proxy for disease mechanisms are increasingly evident. Pseudo-temporal ordering is a method that arranges patients along a continuous molecular axis based on similarity of gene expression profiles. It was initially developed for microarray data^19^, and is now widely applied in single-cell genomics, providing an alternative framework by arranging cells or patients along trajectories based on their molecular states. A recent application of this approach to microarray data from patients with MASLD^20^, suggests that it offers a promising, histology-independent method for investigating MASLD progression via transcriptomic profiles. By complementing traditional assessments, pseudo-temporal ordering provides a data-driven perspective on the molecular changes underpinning disease progression.

In this study, we introduce a framework that uses such patient pseudo-temporal ordering to treat MASLD not as a set of discrete stages but as a continuous molecular process. Using cross-sectional transcriptomic data, we (1) order patients along a data-driven disease axis; (2) identify regulatory programmes that change progressively along this axis; and (3) anchor these changes to circulating biomarkers that enable non-invasive positioning of patients. This structure enabled the interpretation of disease mechanisms, biomarkers and therapeutic hypotheses within a unified progression model.

## Results

### Transcriptomics-based disease trajectory analysis captures MASLD progression

We first sought to determine whether MASLD progression could be represented as a continuous molecular process using liver transcriptomic data alone. Specifically, we asked whether patients could be ordered along a disease axis that recapitulates histological severity while providing finer resolution than discrete staging. Given variability between patients, including the role of sex^21^, comorbid conditions^22^ and other parameters in the timing of disease progression^23^, we focused on modelling the molecular underpinnings of progression that are directly related to MASLD histological phenotypes, as these are common across all patients.

To test this hypothesis, we analysed RNA-seq data from 136 patients across two published cohorts^9,24^, adjusting for sex during data integration (Methods and Supplementary Tables 1 and 2).

**Fig. 1:**
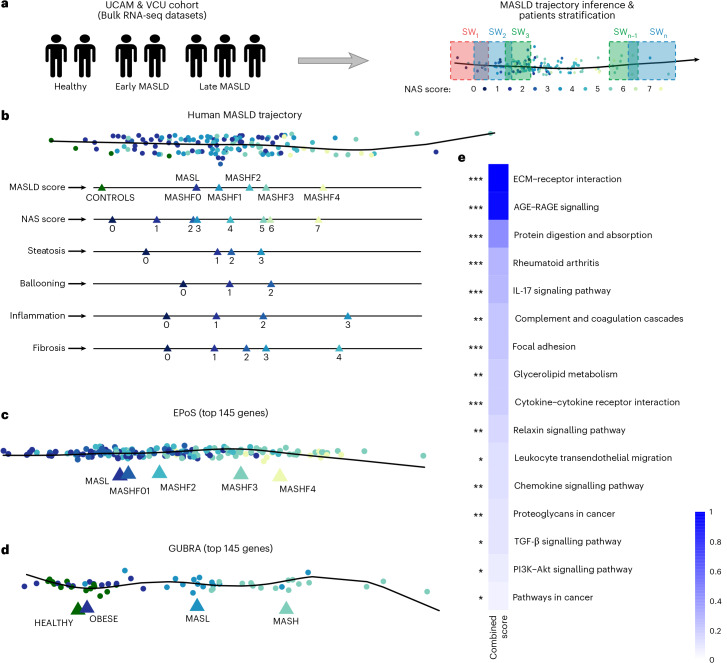
Pseudo-temporal ordering of patients captures disease progression and identifies MASLD gene signature. **a**, Schematic of pseudo-temporal ordering of patients based on bulk transcriptomics data from liver biopsies and stratification of patients into SWs. **b**, Pseudo-temporal ordering of patients based on transcriptomic data recapitulates disease progression based on individual phenotypes (steatosis, ballooning, inflammation and fibrosis), and based on the NAS and MASLD scores. **c**,**d**, Pseudo-temporal ordering of two independent, orthogonal datasets, based on the 145 genes that are most predictive of the trajectory, provides a linear and clear separation of the disease stages (EPoS dataset (**c**) and Gubra dataset (**d**)). Triangles show the average position of each histopathologically characterized stage on the trajectory. **e**. Functional enrichment analysis results of these 145 genes using EnrichR. Significance was assessed using a two-sided Fisher’s exact test, with *P* values adjusted for multiple testing using the FDR method. The combined score (*c*) has been calculated as *c* = log(*P*) × *z*-score, with *z*-score reflecting the deviation from the expected rank. Source data

**Extended Data Fig. 1:**
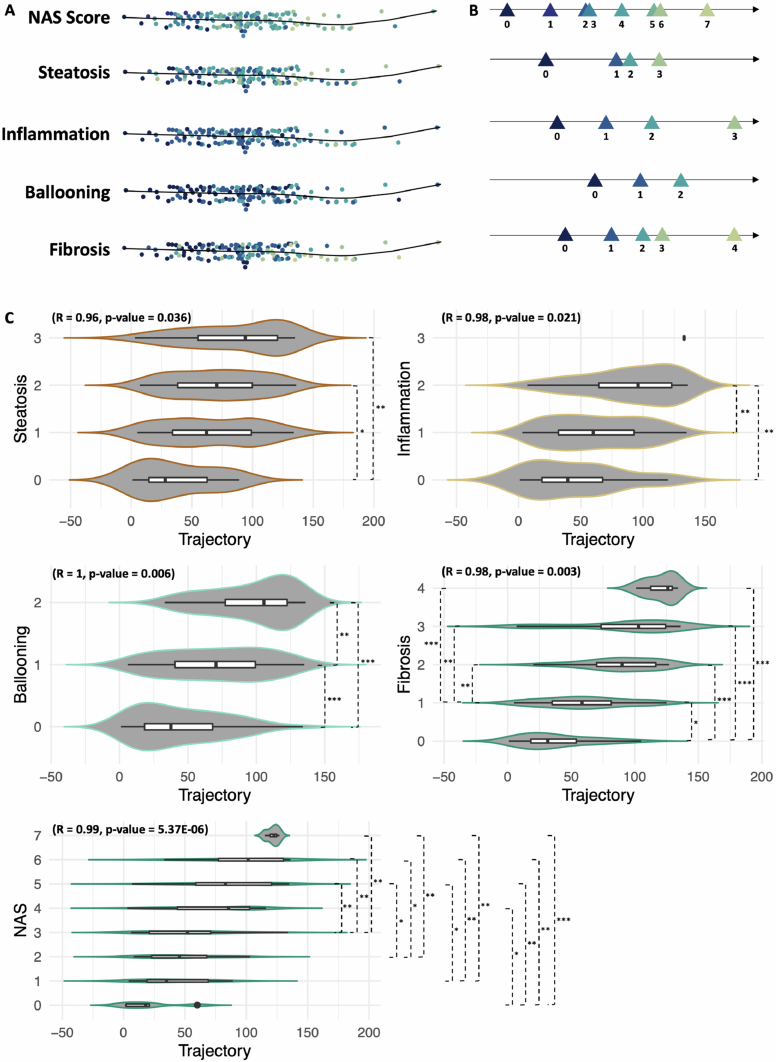
MASLD patients along the continuous transcriptomics-driven trajectory for the internal UCAM-VCU dataset. **A**. The colouring is based on the histopathologists’ scores. **B**. Triangles showing the average horizontal position of each scoring group on the trajectory. Both panels provide information for the NAS score (ranging from 0 to 7), Steatosis scoring (0-3), Inflammatory damage (0-3), Ballooning (0-2), and Fibrosis (0-4). **C**. Violin plots illustrate the distribution of the patient trajectory positions (biological unit of analysis; no technical replicates) within each histopathological scoring group. Group sizes correspond to the number of patients in each histological category and are as follows: Steatosis (0: n = 12, 1: n = 46, 2: n = 54, 3: n = 23); Inflammation (0: n = 13, 1: n = 86, 2: n = 35, 3: n = 1); Ballooning (0: n = 45, 1: n = 56, 2: n = 34); Fibrosis (0: n = 32, 1: n = 49, 2: n = 23, 3: n = 24, 4: n = 7); NAS (0: n = 5, 1: n = 8, 2: n = 14, 3: n = 26, 4: n = 36, 5: n = 32, 6: n = 10, 7: n = 4). Embedded boxplots show the median (centre line), interquartile range (box), and whiskers extending to 1.5x the interquartile range. Each panel shows the Pearson correlation coefficient (R score) between the mean trajectory position of each scoring group. Statistical significance was assessed using one-way ANOVA followed by two-sided Tukey’s honestly significant difference (HSD) post hoc test; multiple comparisons adjusted within the Tukey procedure. The statistically significant groups are indicated with dashed lines (*p-value < 0.05, ** p-value < 0.005, *** p-value < 0.0005; exact p-values and F-statistics are provided in the corresponding source data file). Source data

After excluding one outlier, we applied pseudo-temporal ordering to derive a transcriptomics-based disease trajectory (Fig. 1a, b and Extended Data Fig. 1a,b). The inferred trajectory showed strong concordance with histological measures, including steatosis, ballooning, inflammation, fibrosis and NAFLD activity score (NAS) (Pearson *R* = 0.96–1.0; *P* < 0.05 for most compared disease stages; analysis of variance followed by Tukey’s pairwise comparisons; Extended Data Fig. 1c), confirming alignment with established disease stages. Because the trajectory was inferred from bulk liver transcriptomes, it reflects the tissue’s overall molecular state and therefore captures both cell-intrinsic regulatory changes and shifts in cellular composition that accompany disease progression.

**Extended Data Fig. 2:**
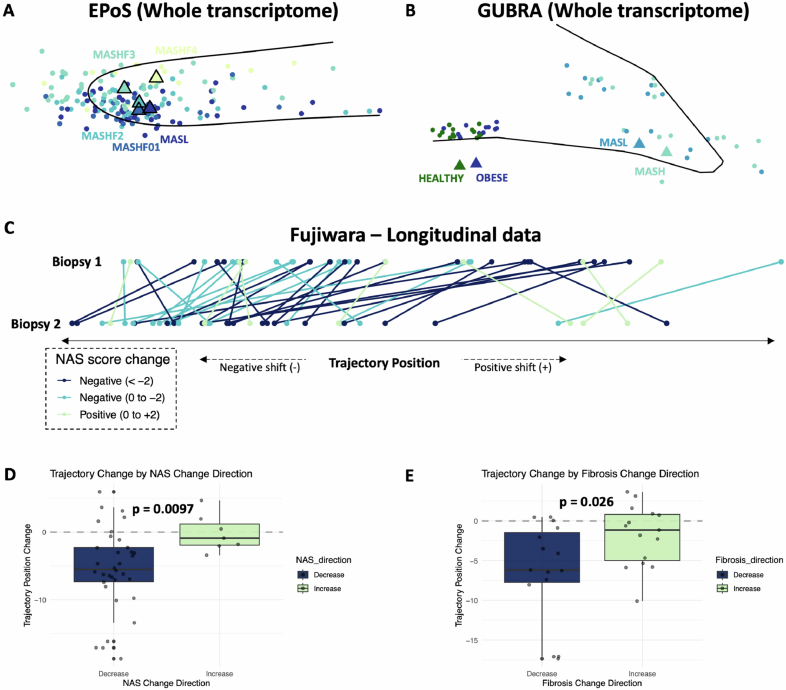
Trajectory inference on independent MASLD transcriptomic datasets and MASLD patients along the continuous transcriptomics-driven trajectory for the external longitudinal (Fujiwara) dataset. **A**. Trajectory inference for the independent EPoS dataset using the whole transcriptome. Colour coding depicts MASLD stages as published with the original RNA-Seq data. Triangles show the average horizontal position of each scoring group on the trajectory. **B**. Trajectory inference for the independent GUBRA dataset. The population was characterized as Healthy (non-obese individuals without MASLD/MASH), Obese (healthy but obese individuals without MASLD/MASH), MASL, and MASH **C**. Fujiwara dataset: Each point represents an individual patient with paired biopsies (independent patients as biological units; no technical replicates). Only patients with a change in NAS score (ΔNAS ≠ 0; n = 41) were included (ΔNAS = NAS_2_ - NAS_1_). Patient movement along the trajectory reflects the change in inferred disease position between the two biopsy measurements. Movement towards the left or right indicates disease regression (ΔNAS < 0) or progression (ΔNAS > 0), respectively. The colour shows the change in NAS score between the two biopsies, with positive scores indicating an increase in the second biopsy. **D**. Distribution of trajectory position shifts stratified by change in NAS score between biopsies (ΔNAS > 0: n = 7; ΔNAS < 0: n = 34; patients with ΔNAS = 0 excluded from group comparisons). Statistical significance of the patients’ position change between increased and decreased NAS score was assessed using a two-sided Wilcoxon rank-sum test (Mann-Whitney U test; *P* = 0.0097). Boxplot shows the median (centre line), interquartile range (box), and 1.5xIQR whiskers, and values beyond the whiskers shown as outliers. **E**. Similar to (D) but for the change in fibrosis score (ΔFibrosis > 0: n = 15; ΔFibrosis < 0: n = 16; patients with ΔFibrosis = 0 excluded). Statistical significance was assessed using a two-sided Wilcoxon rank-sum test (*P* = 0.026). Boxplot definition as in (D). All statistical tests were two-sided. Source data

To validate the trajectory model, we tested it on two independent RNA sequencing (RNA-seq) datasets: the EPoS dataset, a large multi-cohort dataset encompassing 168 patients with MASLD across the full disease spectrum, and the Gubra dataset, comprising 26 healthy individuals with healthy weight and obese individuals, and 31 patients with MASLD and MASH^8,25^. Initial pseudo-temporal ordering of these patients showed a trend of disease progression; however, additional variance in both datasets was observed that did not fully correlate with disease progression (Extended Data Fig. 2a,b).

To identify the principal drivers of variance along the MASLD–MASH trajectory and assess their generalizability, we applied a random forest approach to the discovery cohorts (UCAM/VCU), identifying 145 gene transcripts predictive of disease stage and histopathological features (area under the curve (AUC) 0.62–0.73; Supplementary Table 3). This gene set corresponding to the identified transcripts more accurately recapitulated the disease trajectory across independent datasets^8,25^, improving linearity and stage separation compared with the full transcriptome (Fig. 1c, d). Feature selection was performed exclusively on the discovery data, ensuring no information leakage and supporting the model’s robustness across the larger, more heterogeneous EPoS and Gubra cohorts.

Finally, we evaluated the ability of our 145-gene signature to place longitudinal data from 58 patients of a different ethnic background (the Japanese cohort^26^) along the trajectory. We found that their location along the trajectory was largely consistent with changes in their histological profile, particularly where their disease regressed (Extended Data Fig. 2c–e). Together, these results demonstrate the generalizability of our 145 genes for MASLD trajectory inference.

The identified genes were enriched in pathways central to MASLD–MASH progression, including extracellular matrix (ECM)–receptor interaction and focal adhesion (associated with fibrosis), AGE–RAGE and interleukin (IL)-17 signalling (inflammatory responses), and TGF-β and PI3K-Akt signalling (wound healing and metabolic dysregulation). Other pathways, such as glycerolipid metabolism and cytokine–receptor interactions, reflect the metabolic and immune dysregulation characteristic of MASLD–MASH (Fig. 1e and Supplementary Table 4).

To capture gradual changes along the disease progression axis and gain deeper insights into the molecular changes driving MASLD progression, we divided patients into overlapping groups (‘sliding windows’; SWs), allowing us to detect progressive molecular shifts without imposing discrete stage boundaries. SWs can be viewed as moving windows along disease progression, analogous to smoothing a time series, enabling the detection of early, transient or delayed molecular events that are missed by discrete staging.

**Extended Data Fig. 3:**
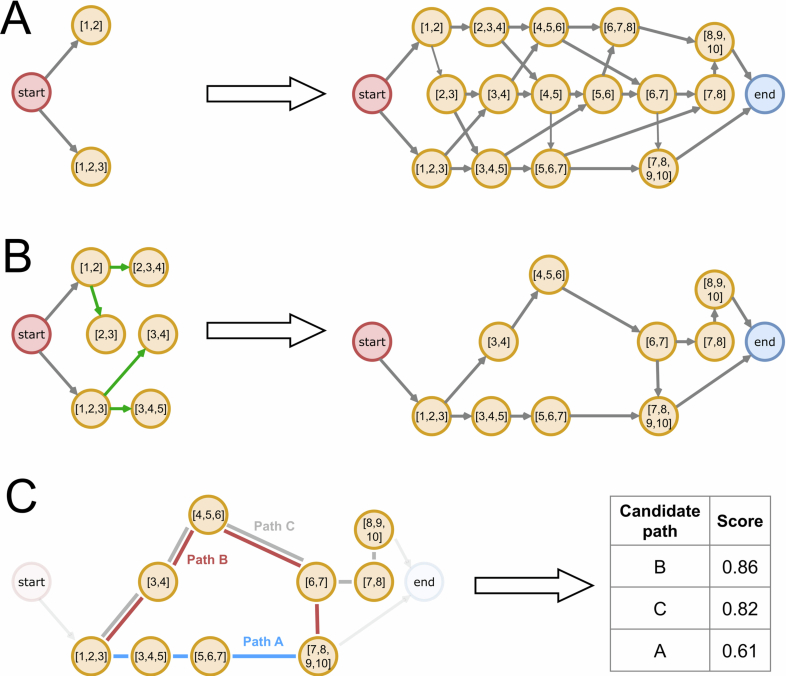
Graph-based approach to find the optimal sequence of sliding windows for the stratification of patients. **A**. Generation of a directed acyclic graph, where each node corresponds to a group of samples (sliding window; SW) aligned by pseudo-temporal order. Directed edges connect overlapping windows, ensuring continuity along the trajectory. The graph is generated iteratively until all the samples have been mapped to SW nodes. **B**. Pruning of the graph to identify candidate SW sequences: differential expression analysis is performed for each linked pair of SWs on the graph, retaining only edges that meet specific criteria regarding the number of differentially expressed genes (FDR < 0.1). **C**. The optimal SW sequence is defined as the one that maximizes the product of three max-scaled criteria: (i) median DEG count, (ii) number of edges exceeding the lower DEG threshold, and (iii) coefficient of variation of DEG counts along the path.

To optimize the SW sequence, we developed a graph-based method that maximized the information content of each window (Methods). Using this approach, patients were divided into 13 groups along the trajectory for functional network analysis (Methods, Fig. 1a, Extended Data Fig. 3 and Supplementary Tables 1 and 5).

### Data-driven global MASLD/MASH network recapitulates key molecular mechanisms of disease

To organize the diverse molecular changes observed along the MASLD trajectory into an interpretable structure, we constructed a MASLD regulatory network that integrates coexpression modules, transcription factor activity and upstream signalling pathways.

**Fig. 2:**
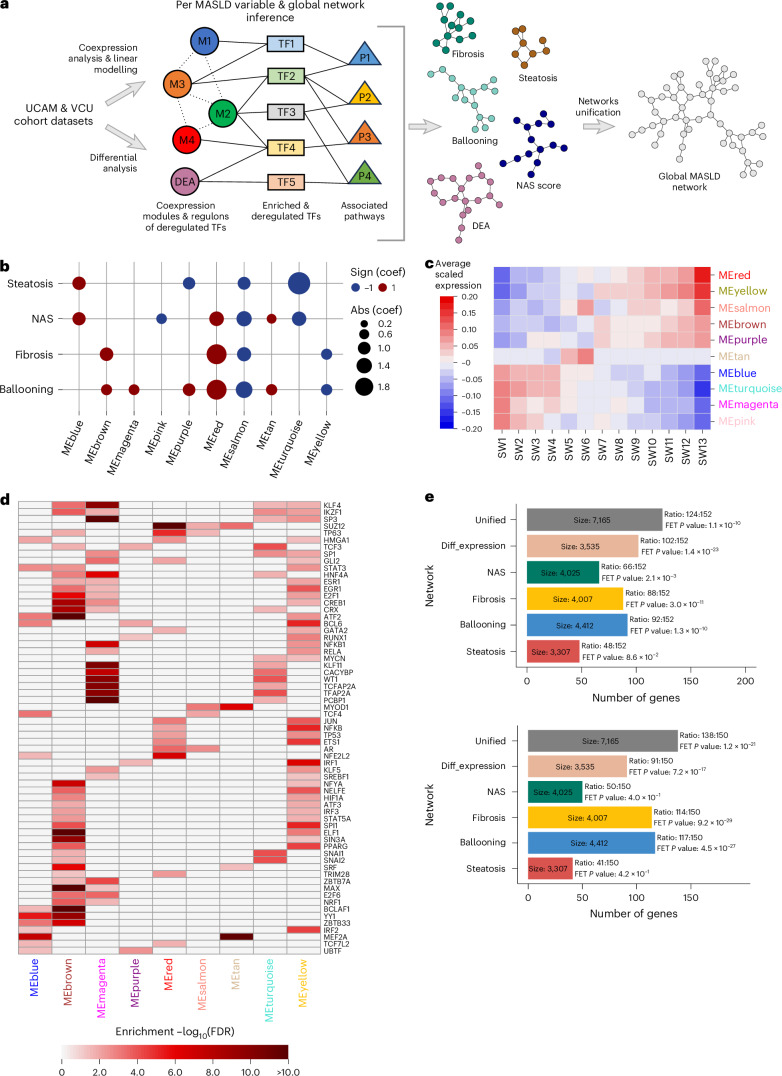
Phenotype-specific gene modules associated with the different MASLD variables. **a**, Schematic of the approach to extract a reference MASLD network. **b**, Association (coefficient from a multivariate linear model) of the different gene coexpression modules with NAS score, steatosis, ballooning and fibrosis. Red indicates a positive coefficient and blue a negative coefficient. These coefficients represent the contribution of each module to the model and should not be interpreted as the direction of module expression changes along the pseudo-temporal trajectory shown in **c**. **c**, The heatmap shows the average scaled expression of the modules along the pseudo-temporal trajectory. The values for each module were calculated by averaging the scores of SW-grouped samples along their first principal component (eigengene). **d**, TFs whose regulons are enriched in the modules. The figure shows TFs per module, coloured according to the adjusted *P* value of the enrichment test. The presented TFs have been identified as enriched in multiple modules. Bold indicates TFs that are significantly deregulated (FDR < 0.05) in at least one SW (see SW analysis results). The complete list of TFs derived from the enrichment analysis is given in Supplementary Table 8**. e**, Enrichment of MASLD network and its components in known MASLD genes curated from the literature (top) or from MSigDB (bottom). Odds ratios were assessed using a one-sided Fisher’s exact test. Source data

First, we adapted our previously published method for generating phenotype-specific networks by integrating paired transcriptomics and phenotype data^27^ (Fig. 2a and Methods). Using weighted gene coexpression network analysis (WGCNA)^28^, we identified gene coexpression modules and linked them to key phenotypic features, including the NAS score, steatosis, ballooning and fibrosis (Fig. 2b and Supplementary Table 6). The ballooning score reflects hepatocyte injury, whereas the inflammation score reflects lobular immune infiltrates. The inflammation score was used as a covariate in the analysis, due to difficulty deconvolving its role as a cause versus effect and because it dominated the signal. Nonetheless it is already represented in the NAS score. Finally, to extract the modules associated with our histological phenotypes of interest, we used linear regression, identifying ten modules associated with at least one phenotypic feature (Fig. 2b and Methods). These modules were selected as significant predictors (non-zero coefficients, false discovery rate (FDR) < 0.05) for the phenotypic features, so the coefficient signs reflect their role in the models, not necessarily the direction of their correlation. For example, fibrosis increases with MEbrown and MEred, whereas MEsalmon and MEyellow adjust the prediction through their negative coefficients, although all four modules are positively correlated with fibrosis along the disease trajectory (Fig. 2c). After filtering genes that were not correlated with the eigengene of each module (Methods), the number of genes in each significant module ranged from 192 in the MEsalmon to 2,949 in the MEturquoise module (Supplementary Table 6).

Reactome^29^ enrichment of the phenotype-associated modules highlighted patterns consistent with MASLD progression (Supplementary Table 7). MEbrown, associated with ballooning and fibrosis, showed robustly strong enrichment for protein translation (R-HSA-72766, FDR = 1.9 × 10^−99^) and ribosome biogenesis (R-HSA-72706, FDR = 5.5 × 10^−59^), reflecting hepatocyte stress and increased biosynthetic demand during injury^30^. MEred, also linked to fibrosis and ballooning, was enriched for ECM organization (R-HSA-1474244, FDR = 6.5 × 10^−23^), aligning with well-established fibrogenic remodelling^31^. In contrast, MEblue, associated with steatosis and NAS, was enriched for gene-regulatory programmes (R-HSA-74160, FDR = 1.6 × 10^−18^) consistent with early metabolic and transcriptional reprogramming in lipid-laden hepatocytes^32^. Finally, MEyellow, associated with fibrosis and ballooning, showed strong enrichment for immune system pathways (R-HSA-168256, FDR = 1.8 × 10^−47^), capturing the inflammatory processes characteristic of advanced disease. Together, these phenotype–pathway correspondences provide confidence that the reference network recapitulates key molecular processes underlying steatosis, hepatocellular injury, inflammation and fibrosis in MASLD.

As a confirmation that our modules are following the expected dynamics reflected in the histology and the underlying biological processes, we evaluated the ‘activity’ of the modules along our SWs identifying two groups. Modules that work as predictors for all phenotypic features and are generally associated with ECM organization (MEred and MEsalmon) and inflammatory response (MEyellow and MEpurple) were less active in the beginning of the trajectory and peaking at later stages (Fig. 2b, c). In contrast, modules that are mainly associated with steatosis and NAS, with MEblue and MEpink also strongly related to metabolism, behave oppositely. Of note, MEtan, which acts as a positive predictor of ballooning and NAS, shows activity only in the early–mid stages (SW5–7), in agreement with histology.

Transcription factor (TF) enrichment analysis of the phenotype-associated modules identified 199 TF regulons across nine out of the ten significant modules (FDR < 0.05), with many converging on TGF-β-driven fibrogenic signalling (Supplementary Table 8). Among these, 65 TFs were shared across multiple modules (Fig. 2d). Core TGF-β regulators SMAD2, 3 and 4, were selectively enriched in the MEred module (FDR = 0.04, 5 × 10^−5^, 6.3 × 10^−8^ respectively; Supplementary Table 8), consistent with its link to ballooning and fibrosis (Fig. 2b). Enriched in the ballooning and fibrosis-associated modules, we found SP1, SRF and ETS1, known to interact with TGF-β signalling to promote the expression of tissue remodelling and fibrosis factors^33,34^. EGR1, a TGF-responsive TF previously linked to both steatosis and fibrosis^35^, was enriched across multiple modules, bridging early and late disease features (Fig. 2d).

HNF4A, a key regulator of liver development and morphogenesis^36^ and PPARG, which controls lipid storage and adipocyte differentiation in liver^37^, appeared across modules with opposing phenotype associations (MEbrown versus MEturquoise or MEyellow; FDR HNF4A = ~0.001, PPARG = 1 × 10^−4^ and 4.3 × 10^−^^5^; Fig. 2b,d), indicating divergent transcriptional programmes across disease stages. SREBF1, found in MEyellow (FDR = 0.05) and MEmagenta (FDR = 0.04), similarly bridges lipogenic regulation and early stress responses, consistent with its shift from metabolic control to activation under hepatocellular injury^24,38^.

Inflammatory and hypoxia-responsive TFs (NFKB1, RELA and HIF1A) were enriched in MEyellow (FDR = 6.8 × 10^−^^11^, 9.6 × 10^−^^11^ and 0.006, respectively), whereas CREB1 showed the strongest enrichment in MEbrown (FDR = 2.0 × 10^−^^24^), reflecting distinct immune-driven^39–41^ versus hepatocyte-intrinsic^42^ stress transcriptional contexts (Fig. 2b,d and Supplementary Table 8).

To ensure comprehensive regulatory coverage, we additionally incorporated TFs differentially regulated along the disease trajectory (FDR < 0.05; Methods), yielding a combined TF module (DEA), in which 85 of the 199 enriched TFs showed stage-specific deregulation (Supplementary Table 8).

We then integrated the phenotype-associated modules, their enriched TFs and corresponding pathways (Supplementary Table 9 and Methods) to construct module-specific regulatory networks for each histological feature, as well as for our dynamically regulated TFs along the disease trajectory (Fig. 2a). These networks were then merged into a comprehensive MASLD–MASH disease network, henceforth referred to as the MASLD network for simplicity, to capture dynamic changes in cellular processes across disease stages (Fig. 2a, Methods and Supplementary Table 10). The resulting network contained 7,165 nodes and showed significant enrichment for previously reported MASLD-associated genes from both our curated literature set (Supplementary Table 11) and an established MSigDB^43^ gene set (MSigDB ID M39806; Methods and Fig. 2e).

As an orthogonal validation we used the same pipeline to generate a MASLD network using the larger EPoS dataset described above^8,25^. That network comprised 6,732 nodes (Supplementary Table 10) and the intersection of the two networks was 5,241 nodes (Jaccard index = 0.61, odds ratio = 7.11, Fisher’s exact test *P* = 0).

### Sliding window analysis highlights molecular dysregulation in MASLD progression

We next sought to combine our reference MASLD regulatory network with our patient trajectory using our SW approach (Fig. 1a) to explore the molecular mechanisms underlying MASLD progression relevant to observed histological phenotypes.

**Fig. 3:**
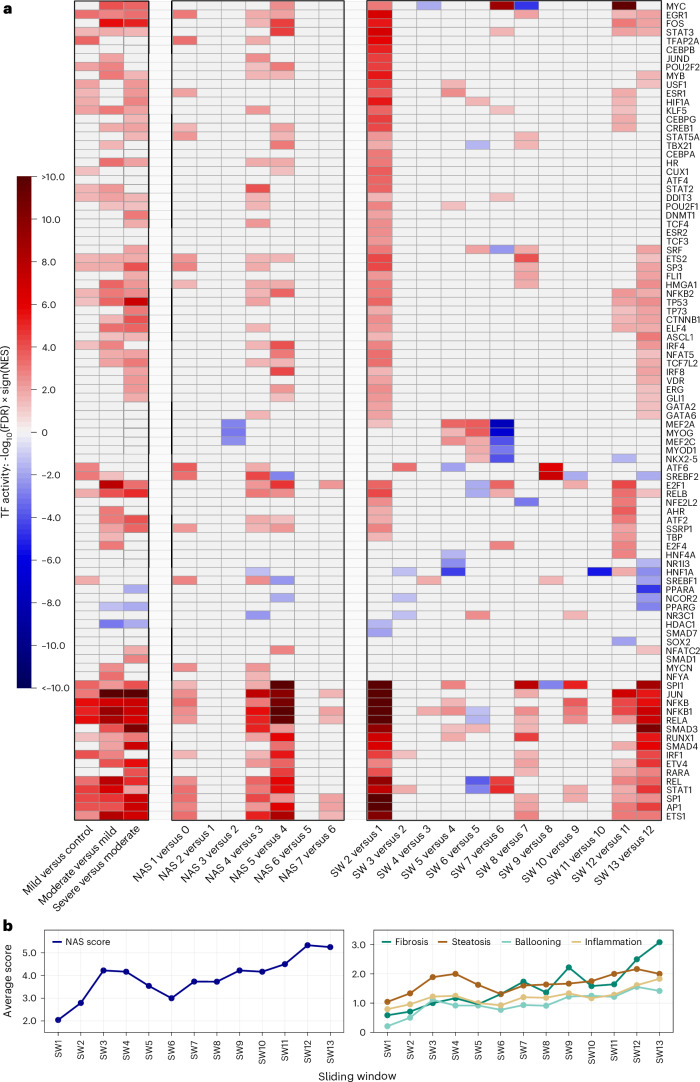
Sliding window analysis to study the landscape of molecular changes along the MASLD trajectory. **a**, Results from TF activity analysis in two types of discrete patient stratification (mild, moderate, severe and NAS scores), and the pseudo-temporal trajectory. The colour indicates a change in TF activity compared with the previous disease stage. Only results with FDR < 0.01 are shown. **b**, Mean phenotype and NAS pathologist scores along the SW trajectory. Source data

**Extended Data Fig. 4:**
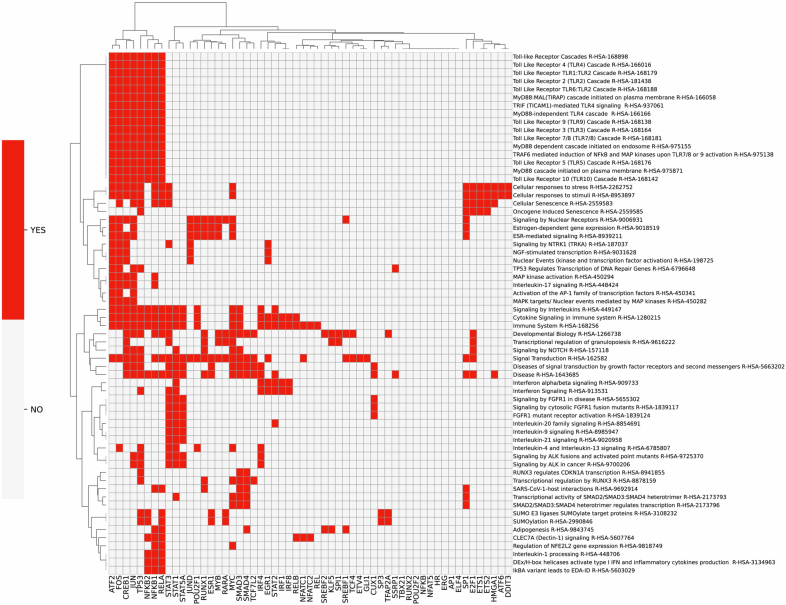
Agglomerative clustering of the 57 TFs that were found to be deregulated in two types of discrete patient stratification and the pseudotemporal trajectory (FDR < 0.05). Reactome pathway enrichment analysis (Fisher’s exact test, Benjamini–Hochberg, FDR < 0.001) was performed to link TFs to molecular pathways and to create the corresponding one-hot encoding matrix. The clustering was performed using this matrix to identify groups of functionally related TFs. Source data

Initially, we identified TFs that were dynamically regulated across MASLD progression, showing strong concordance with traditional stage-based stratifications (early, middle, late and NAS score-based patient stratification), while providing greater temporal resolution (Fig. 3a and Supplementary Table 12). In total, 122 TFs exhibited at least one significant deregulation event along the trajectory (FDR < 0.05), of which, 57 were also detected by mild–moderate–severe or NAS-based analyses with concordant directionality. This shared set included TF clusters enriched for Toll-like receptor signalling (for example FOS, JUN, CREB1, TP53, NFKB1/2 and RELA; R-HSA-168898, FDR = 5.3 × 10^−5^), oestrogen receptor-mediated signalling (for example ESR1, RUNX1 and MYB; R-HSA-8939211, FDR = 6.4 × 10^−^^7^) and cytokine signalling (for example EGR1, IRF and STAT family members; R-HSA-1280215, FDR = 2.8 × 10^−8^; Extended Data Fig. 4).

SREBF1 was consistently upregulated in early and intermediate disease states across all stratifications (in SW4 and SW9 according to the SW approach), confirming its pro-steatotic role^24,44^. However, only the trajectory-based and NAS-based approaches captured its downregulation in later stages^45^, illustrating the benefit of increased granularity.

Beyond this shared signal, the trajectory-based approach uniquely identified several key regulatory events missed by discrete classifications, including early downregulation of HNF4A (SW5), NR1I3 (SW5) and HNF1A (SW3 and SW5), reflecting disrupted hepatocyte metabolic identity, inflammatory signalling and tissue remodelling^46–48^ (Fig. 3a). Conversely, sustained upregulation of pro-fibrotic regulators such as SRF^49^ and dynamic regulation of TGF-β pathway components, including downregulation of the inhibitory SMAD7 (ref. ^50^) (SW2), were detected only along the SW trajectory (Fig. 3a). Additional TFs (for example MYC, STAT1 and NF-κB1) displayed complex, stage-dependent regulation, underscoring the ability of trajectory-based analysis to capture nuanced and biologically meaningful regulatory dynamics during MASLD progression (Fig. 3a).

**Extended Data Fig. 5:**
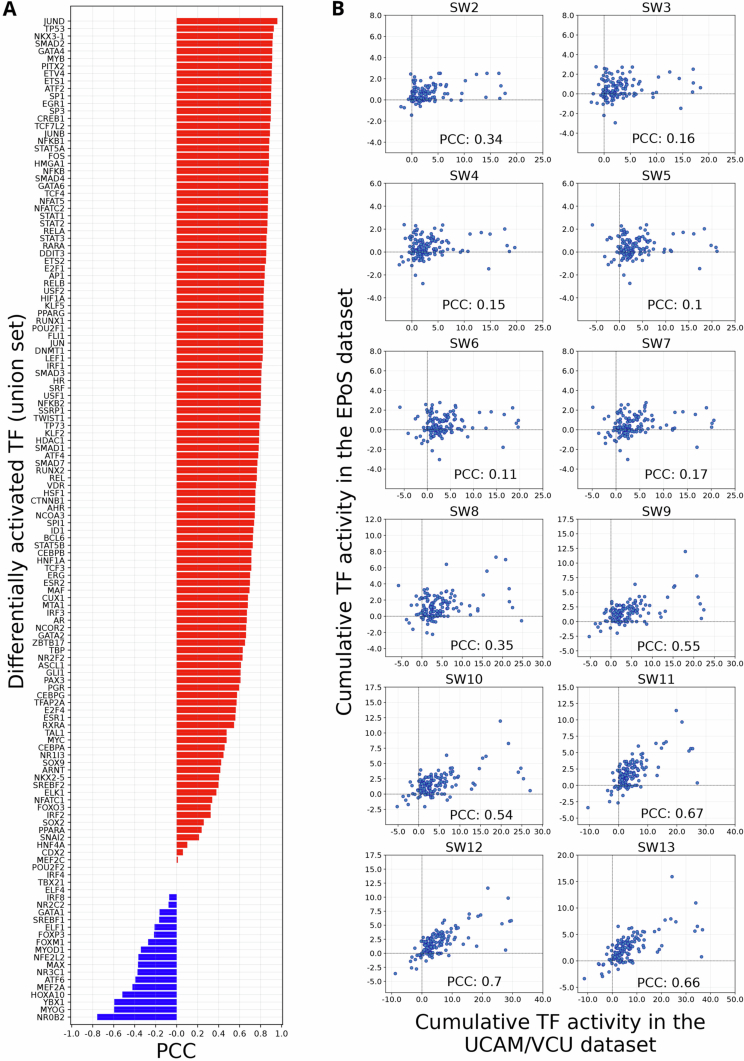
Correlation plots of the activities of TFs for the UCAM/VCU and EPoS datasets. **A**. Pearson correlation of cumulative activation scores of TFs, which were identified as deregulated (FDR < 0.05) based on the sliding windows analysis in at least one dataset. **B**. Pearson correlation of the cumulative TF activation scores between the two datasets for each sliding window. Each dot represents a TF identified as deregulated (FDR < 0.05) at least in one sliding window of a dataset. Source data

These results were largely validated in an orthogonal analysis of the EPoS dataset^8,25^, where 69 TFs were deregulated in at least one SW, 57 of which overlapped with our primary analysis (Jaccard index = 0.43, odds ratio = 76.03, Fisher’s exact test *P* < 2.2 × 10^−16^). Deregulation directionality, based on cumulative activation scores, showed high concordance between datasets with an average Pearson correlation coefficient (PCC) of 0.56 (Extended Data Fig. 5a). The average PCC of cumulative activities per SW was 0.38, revealing two opposing trends along the disease trajectory (Extended Data Fig. 5b). Notably, PCC decreased monotonically in early stages (SW2–SW5), indicating divergence in early molecular profiles between cohorts, likely due to the absence of healthy samples and higher MASLD variable values in SW1 of the EPoS dataset, which makes this group more similar to SW2 than in the UCAM/VCU cohort. In contrast, PCC increased in middle and late stages, demonstrating that despite cohort-specific differences, the trajectory-based approach captures consistent molecular programmes as disease progresses (Extended Data Fig. 5b).

An intriguing cluster of TFs, including MEF2A, MEF2C, MYOG, NKX2-5 and MYOD1, exhibited unique activity patterns in our trajectory analysis that were not detected using traditional discrete patient staging (Fig. 3a). Their activity peaked during SW5–SW6 and then sharply declined at SW7, suggesting either a resolution of activation or a shift in regulatory dynamics. Notably, no significant changes in their activity were observed in later stages, indicating a potential stabilization of their regulatory influence as the disease progressed (Fig. 3a). These TFs, known primarily for their roles in myogenesis^51^, have also been implicated in hepatic stellate cell activation and their transition to a myofibroblast-like phenotype, a critical driver of fibrosis^52^. MEF2A–MEF2C and NKX2–5 also have documented roles in macrophage differentiation and inflammatory programming^53–55^, although not specifically related to MASLD. Further research is needed to understand the role of this TF cluster in MASLD progression.

**Extended Data Fig. 6:**
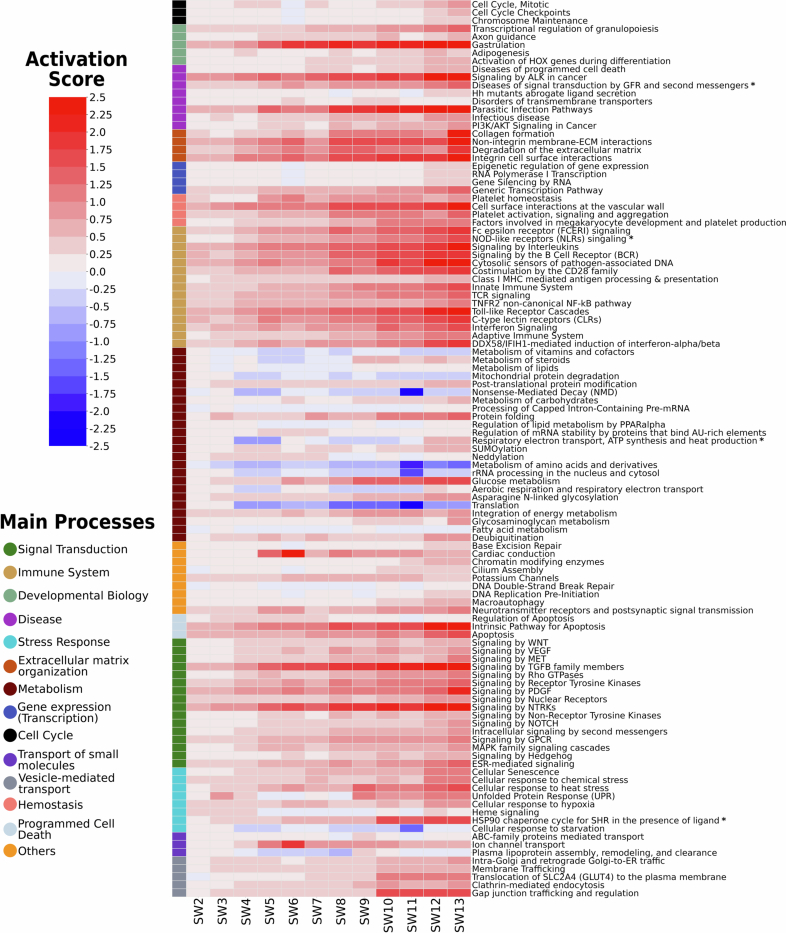
Cumulative activation scores of Reactome pathways along the pseudotemporal trajectory. This list of terms provides a comprehensive description of MASLD-relevant pathways, derived from the enrichment analysis of deregulated network signatures along the disease trajectory and a pruning procedure applied to the hierarchical structure of Reactome. The SW-based activation score of each pathway was defined as the sum of the activation scores of its related genes, normalized by the pagerank centralities of these genes within the pathway-based subnetwork. The respective cumulative scores were defined by progressively summing the SW-based activation scores. Pathway names annotated with “*” have been shortened to fit within the figure width. Source data

**Extended Data Fig. 7:**
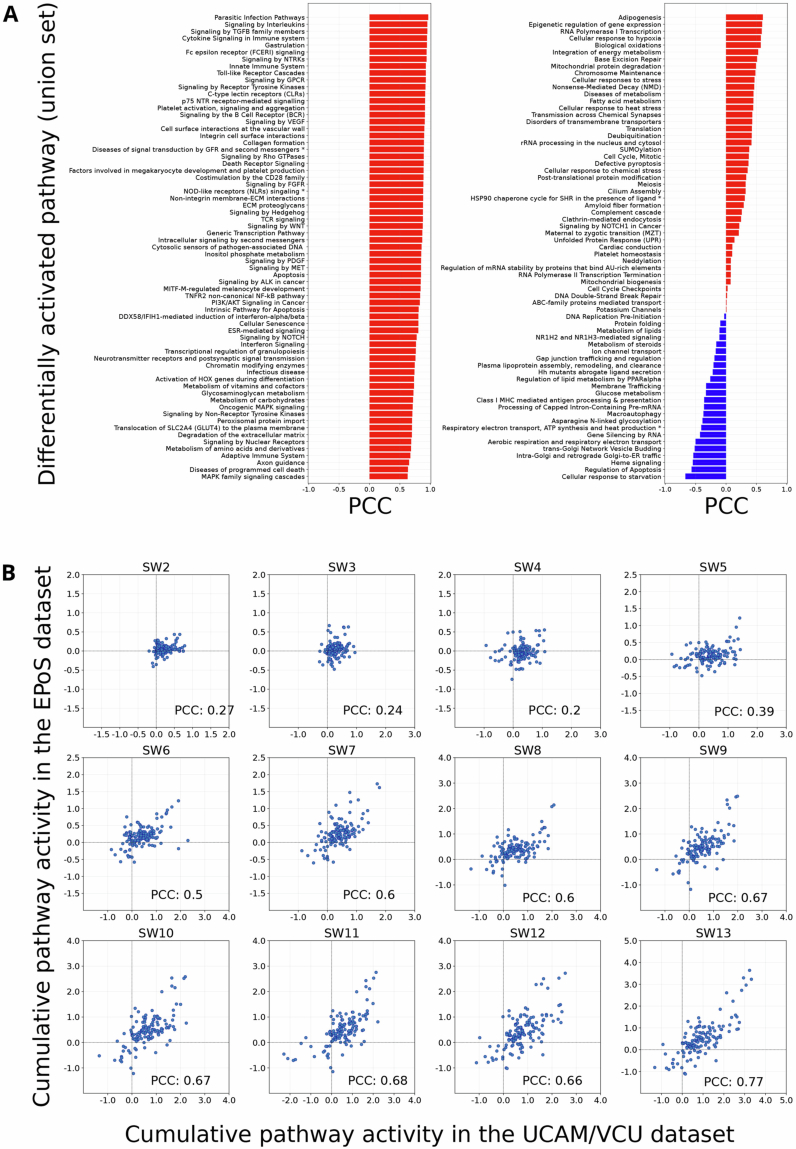
Correlation plots of the activities of pathways for the UCAM/VCU and EPoS datasets. **A**. Pearson correlation of cumulative activation scores of Reactome pathways that were identified as MASLD-relevant, based on the sliding windows analysis in at least one dataset. Pathway names annotated with “*” have been shortened to fit within the figure width. **B**. Pearson correlation of cumulative activation scores of Reactome pathways between the two datasets for each sliding window. Each dot represents a pathway identified as MASLD-relevant at least in one dataset. Source data

To explore the underlying molecular processes, we employed a network propagation-based strategy to extract differentiated network signatures for each SW from the MASLD reference network (Methods). Reactome analysis of these networks identified 111 pathways exhibiting progressive changes along the disease trajectory (Extended Data Fig. 6, Supplementary Table 13 and Methods). Signal transduction, including TGFβ, receptor tyrosine kinase and others, and multiple immune pathways increased with disease progression (Extended Data Fig. 6), consistent with escalating inflammatory and signalling dysregulation. ECM organization pathways, linked to fibrosis, such as integrin and non-integrin membrane-ECM interactions, were upregulated predominantly from mid to late stages (Extended Data Fig. 6). Metabolic pathways showed more complex dynamics: lipid metabolism was initially downregulated (SW3–5) but showed modest upregulation after SW8, whereas glucose metabolism increased persistently from early stages, consistent with insulin resistance^56^. Orthogonal validation in the EPoS dataset^8,25^ identified 95 associated pathways, with 72 overlapping (Jaccard index = 0.54, odds ratio = 7.23, Fisher’s exact test *P* = 6.3 × 10^−12^; Supplementary Table 13). Pathway deregulation directionality was concordant across datasets (average PCC = 0.46; Extended Data Fig. 7a), and cumulative pathway activities per SW showed even higher agreement (average PCC = 0.52; Extended Data Fig. 7b), mirroring oscillatory patterns observed in TF-based analyses but with higher overall correlation.

Finally, we compared pathway dysregulation across SWs with histopathological scores (steatosis, ballooning, inflammation, fibrosis and NAS; Fig. 3b). Although these scores increased overall with disease progression, several pathways showed dysregulation earlier than histological changes. For example, extracellular matrix pathways were upregulated at SW8, attenuating the overall increase in fibrosis observed later along the trajectory, aligned with significant changes in multiple TFs (Fig. 3a) in that stage, indicating that molecular readouts may detect MASLD progression earlier than conventional assessments.

Overall, the enhanced resolution of our sliding-window-based approach provides a comprehensive molecular landscape of MASLD progression, shedding light on the interplay among immune activation, fibrotic remodelling and metabolic dysregulation over time.

### Cell-type deconvolution along the MASLD trajectory reveals network changes associated with tissue composition

Changes in liver cell composition and state are hallmarks of MASLD progression and reflect injury- and inflammation-driven remodelling of the hepatic microenvironment^1^. Characterizing the molecular programmes underlying these shifts can help contextualize disease mechanisms and inform biomarker discovery.

**Fig. 4:**
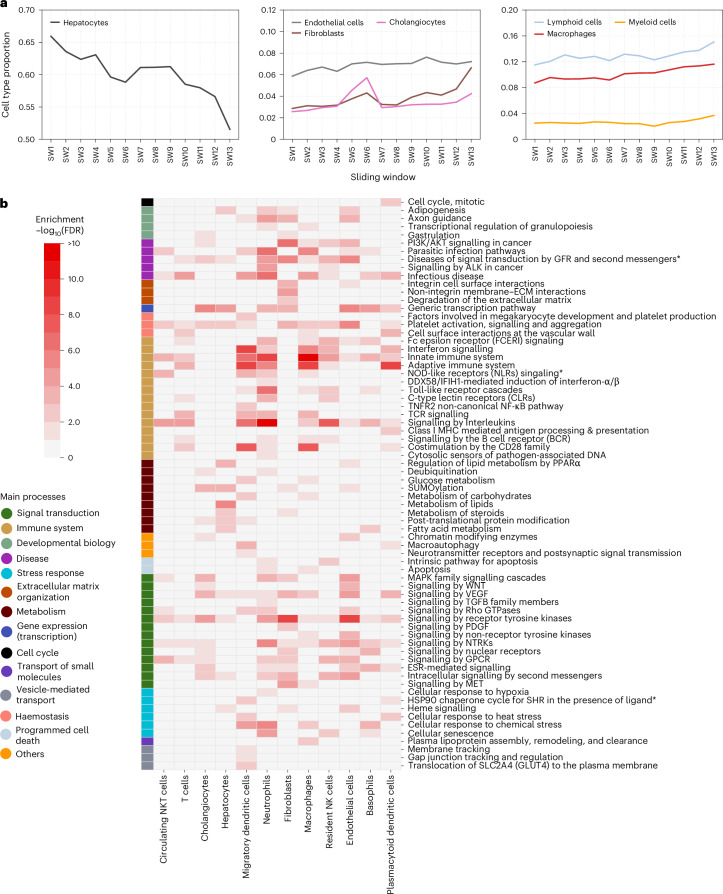
Results of cell type deconvolution analysis. **a**, Cell type deconvolution of patient transcriptomic data along our MASLD trajectory **b**, Prediction of cell types with which the deregulated processes are associated after excluding gene sets of these processes that could have been identified as differentiated by the mere change in abundance of cell types. Only enrichments with *P* < 0.05 (Fisher’s exact test) are shown; pathways without significant enrichment were excluded. Source data

**Extended Data Fig. 8:**
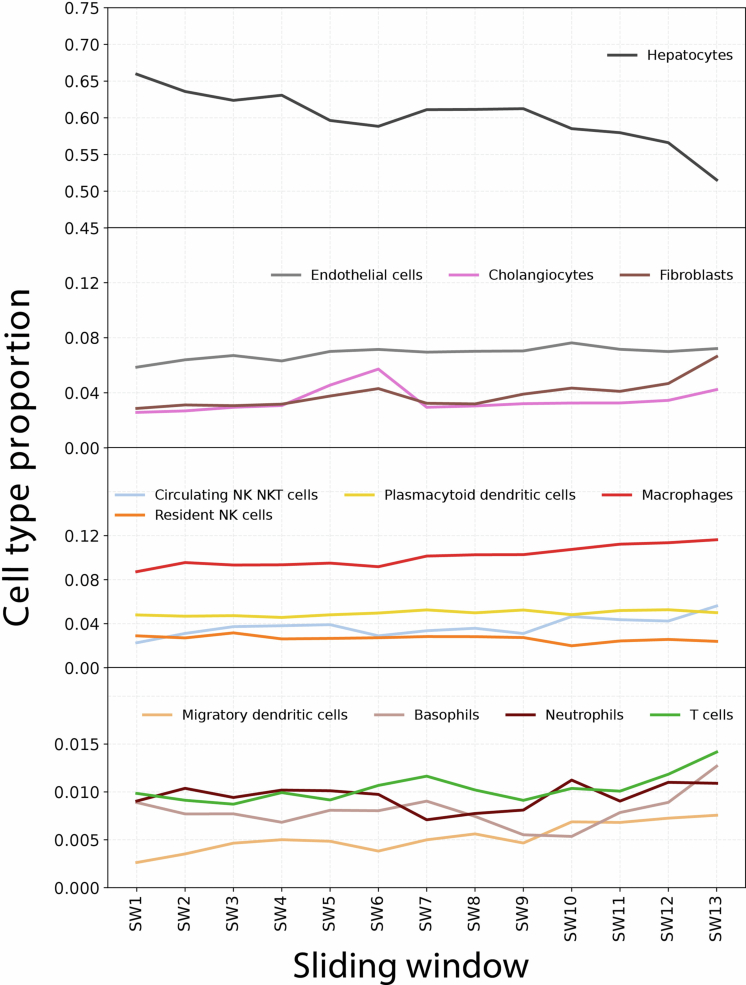
Consensus proportion of cell type along the disease trajectory. Cell type deconvolution was applied to the bulk RNA-seq samples using 6 methods. The average cell type proportions for each sliding window were calculated by averaging the proportions across the corresponding samples. Finally, the consensus result was calculated as the mean of cell-type proportion per cell-type across methods. Cell types with maximum proportion less than 0.5% along the disease trajectory have been excluded. Source data

To characterize cellular dynamics along the trajectory, we performed cell-type deconvolution of bulk liver transcriptomes (Methods, Extended Data Fig. 8 and Supplementary Table 14). Hepatocytes remained the dominant cell type but progressively declined from early to late stages (from 0.66 in SW1 to 0.52 in SW13), consistent with increasing contributions from other hepatic and immune populations. To obtain robust estimates, haematopoietic cells were aggregated into myeloid and lymphoid lineages, excluding macrophages, which showed a distinct and progressive increase, particularly after mid-progression (SW6; Fig. 4a and Extended Data Fig. 8). Lymphoid cells (predominantly T cells and natural killer (NK)/NKT cells) increased more markedly than other myeloid populations, alongside rising cholangiocyte and fibroblast signals, reflecting heightened inflammatory and fibrogenic activity. In this analysis, the ‘fibroblast’ annotation predominantly reflects hepatic stellate cells, which comprise ~70% of this category in the reference single-cell atlas^57^. In contrast, endothelial cell proportions remained relatively stable, suggesting preservation of vascular structure even in advanced disease.

We next linked cell types to deregulated processes along the trajectory. To distinguish true process dysregulation from shifts in cell composition, we removed genes whose expression changes could be explained by changing cell proportions, using pseudo-bulk profiles derived from healthy liver single-cell data (Methods).

Enrichment analysis of the remaining gene sets revealed cell-type-specific pathway associations (Fig. 4b and Supplementary Table 15). As expected, lipid metabolism was primarily hepatocyte-associated, immune pathways mapped mainly to macrophages, neutrophils and migratory dendritic cells, and ECM and fibrotic processes were strongly linked to fibroblasts (Fig. 4b). Non-immune signalling pathways, including Wnt, receptor tyrosine kinase and Rho signalling, were predominantly associated with endothelial cells, with additional contributions from fibroblasts, cholangiocytes and immune cells. Together, these results highlight coordinated crosstalk between parenchymal, stromal and immune compartments during MASLD progression.

### MASLD Trajectory-specific biomarkers through integrated plasma–liver expression analysis

From a clinical perspective, the key question is whether molecular trajectories inferred from liver tissue can be accessed non-invasively and used for patient stratification. We therefore focused on identifying circulating biomarkers that not only predict fibrosis stage but also position patients along the continuous disease trajectory.

**Fig. 5:**
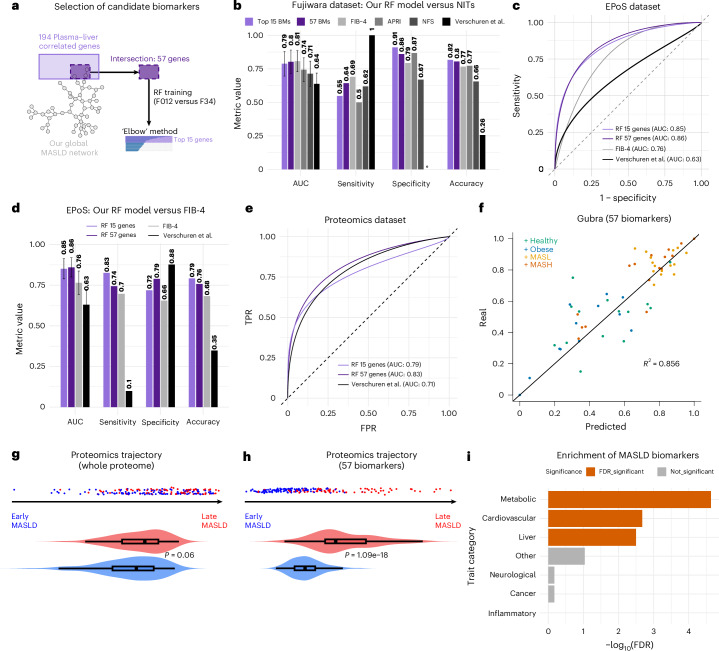
Biomarker selection and performance on the external liver transcriptomics and plasma proteomics datasets. **a**, Selection of candidate biomarkers. A 57-gene set was defined by intersecting the global MASLD network with plasma–liver-correlated genes^58^, followed by random forest classification of fibrosis stage (F0–2 versus F3–4). Feature importance and the elbow method identified a reduced 15-gene subset. **b**, Performance of the 57- and 15-gene classifiers compared with non-invasive clinical scores and a previously published three-gene biomarker panel in the Fujiwara cohort. Both the 57-gene (AUC = 0.8; 95% CI = 0.72–0.89) and the 15-gene (AUC = 0.79; 95% CI = 0.7–0.88) classifiers showed comparable performance with FIB-4 (AUC = 0.81; 95% CI = 0.73–0.89; DeLong test, two-sided *P* = 0.9). Higher AUC values were observed against APRI (AUC = 0.74; 95% CI = 0.65–0.83; *P* = 0.21) and NFS (AUC = 0.71; 95% CI = 0.62–0.8; *P* = 0.06), although these differences were not statistically significant (two-sided DeLong test). In contrast, both models outperformed the three-gene panel (AUC = 0.64; 95% CI = 0.56–0.72; *P* = 0.0004; two-sided DeLong test). 95% CIs are shown for AUC, where the central value represents the AUC estimate and error bars indicate the corresponding 95% CI derived from the receiver operating characteristic (ROC) analysis. AUC is reported as a threshold-independent measure of model discrimination. Sensitivity, specificity and accuracy are reported as point estimates from a single evaluation on independent external cohorts; therefore, no data distribution or error bars are shown for these metrics. **c**,**d**, Benchmarking against FIB-4 and the established three-gene biomarker panel in the EPoS cohort. ROC curves (**c**) and performance (**d**) using additional metrics. Both the 57-gene (AUC = 0.86; 95% CI = 0.8–0.92) and 15-gene (AUC = 0.85; 95% CI = 0.79–0.91) classifiers outperformed FIB-4 (AUC = 0.76; 95% CI = 0.69–0.84) and the three-gene published panel (AUC = 0.63; 95% CI = 0.54–0.72), with statistically significant differences (two-sided DeLong test; *P* = 0.015 and *P* = 0.026 against FIB-4, for both 57 and 15-gene classifiers, respectively; *P* < 0.001 for both versus the three-gene panel). **e**, ROC curve for fibrosis classification of patients using external plasma proteomics data for our 57-gene (AUC = 0.83) and 15-gene (AUC = 0.79) classifier against the published three-gene panel (AUC = 0.71), with statistical comparisons calculated using two-sided DeLong tests; 57-gene versus 15-gene: *P* = 0.03; 57-gene versus three-gene: *P* = 0.00006; 15-gene versus three-gene: *P* = 0.05. Only two proteins (IGFBP7 and SEMA4D) were used for the classification based on the external three-gene panel, providing the closest possible approximation based on their availability and presence in the plasma proteomics dataset (SSC5D was absent). **f**. Random forest regression predicting patient positions along the MASLD trajectory in the Gubra cohort; predicted positions (57 genes) versus transcriptome-derived positions are shown (*R*^2^, Pearson correlation). **g**,**h**, Inferred patient trajectory positions on the external validation plasma proteomics dataset, using either the whole proteome (**g**) or only the 57 biomarkers (**h**). Each point represents an individual patient (biological unit of analysis; no technical replicates). Group sizes correspond to patients in the external proteomics dataset (early MASLD: *n* = 112; late MASLD: *n* = 79). *P* values were obtained using a two-sided Wilcoxon rank-sum test, comparing pseudo-time distributions between early and late disease stages. Boxplots show the median (centre line), interquartile range (IQR) (box) and whiskers extending to 1.5 × IQR. **i**. GWAS trait enrichment of the 57-gene biomarker panel; significant categories after FDR correction are shown in orange (Fisher’s exact test on the biomarkers associated with each of those categories, against the whole GWAS as the background). Source data

Govaere et al.^58^ recently identified 194 genes whose expression correlates between liver tissue and blood plasma across MASLD progression stages. We reasoned that biomarkers derived from this set would be detectable in circulation while still reflecting hepatic molecular processes. To further prioritize markers with mechanistic relevance to disease progression, we focused on the subset of 57 genes that were also present in our MASLD regulatory network, as these genes are directly embedded within disease-associated regulatory programmes. (Fig. 5a and Supplementary Table 1).

**Extended Data Fig. 9:**
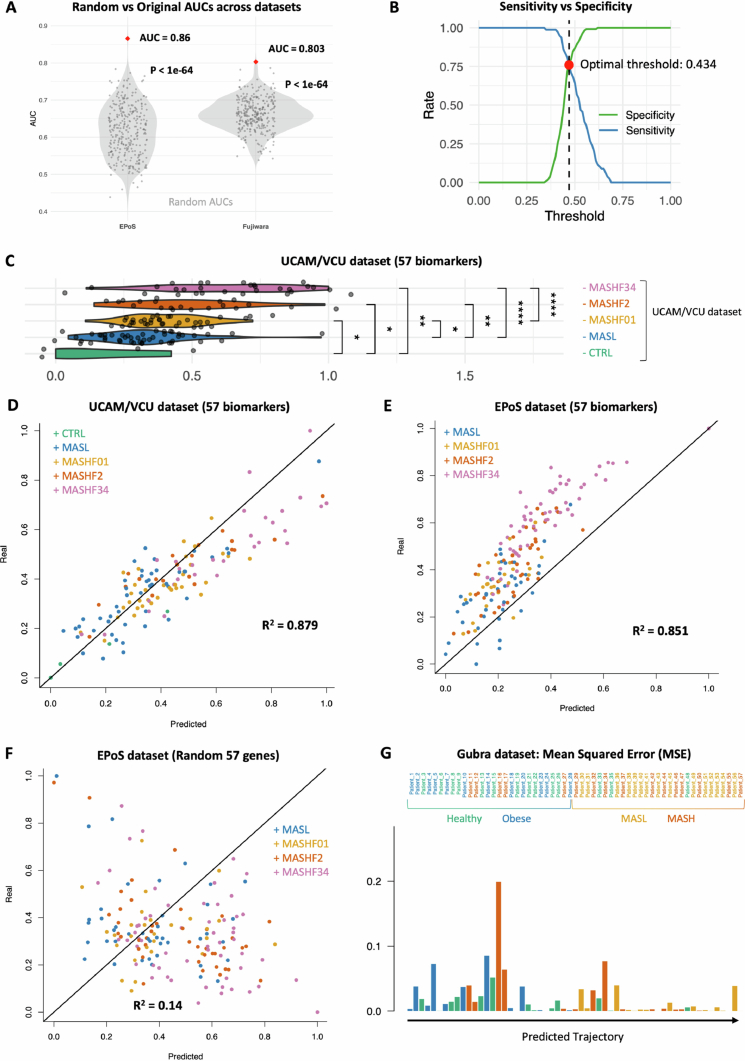
Performance of the random forest classification and regression models. **A**. Model performance using the 57-biomarker set compared to using random genes. In both external validation datasets (EPoS and Fujiwara), similar models were run 300 times, using random 57-gene signatures as input. The red points show our model’s AUC for the 57-biomarker set, while the grey points show the model’s performance for the random sets. The empirical p-values (one-sided) derived from a null distribution generated by random gene sets (n = 300); Null hypothesis: the original 57-gene model performs no better than random 57-gene sets; Method: permutation-based empirical test. **B**. Selection of optimal threshold using Youden’s index on the proteomics dataset. **C**. Patient distributions on the predicted trajectory using the random forest regression model for the internal UCAM/VCU dataset. The analysis was run 1000 times using 5-fold cross-validation and generated the distribution using the mean result for each patient. Different colours denote different MASLD stages. Asterisks represent statistical significance after applying one-way ANOVA (two-sided), followed by Tukey’s post hoc test among the disease stages; *: p < 0.05, **: p < 0.005, ***: p < 0.0005, ****: p < 0.00005. Exact p-values are provided in the corresponding source data file (“SourceData_Extended_figure9.xlsx”). **D**. The y-axis shows the positions of the patients on the “real” trajectory, inferred using the whole transcriptome. The x-axis shows the predicted positions of patients using only the 57 biomarkers. R^2^ denotes the Pearson correlation score for real vs predicted positions on the trajectory. **E**. Similar to (**D**) but for the external EPoS dataset. **F**. Similar to (**E**) but for random 57-gene signatures. **G**. Results of the random forest regression model that sorts patients on the MASLD trajectory and performance on the Gubra dataset (additional validation dataset). Mean squared error for the predicted patients’ positions on the trajectory. The different colours depict the various disease stages (Healthy, Obese, MASL, MASH). The patients are sorted by their positions along the trajectory, independent of their relative distances. Source data

We trained a machine-learning model (random forest classifier) using the 57-gene set to predict fibrosis stage (F0–F2 versus F3–F4), achieving 86.2% accuracy in the UCAM/VCU cohort (AUC = 0.769). Applying the elbow method to feature importance yielded an optimal 15-gene subset, which might be more tractable for clinical application (Fig. 5a and Supplementary Table 1). Validation in two independent cohorts showed robust performance. Specifically, in the Fujiwara cohort^26^, the model achieved an AUC of 0.803 (0.794 for the 15-gene subset), performing similarly or outperforming established clinical scores (FIB-4, AST-to-platelet ratio index (APRI) and NAFLD fibrosis score; NFS) and a three-gene biomarker panel^59^ (Fig. 5b). In the EPoS cohort^8^ the model reached an AUC of 0.86 (0.85 for 15 genes), significantly outperforming the three-gene published biomarker panel (DeLong test; *P* < 0.001) and the FIB-4 AUC (by 10%; *P* = 0.015 and 0.026, respectively, for both models; Fig. 5c), retaining a stronger performance in all evaluation metrics tested (Fig. 5d). Robustness analysis against 300 random 57-gene signatures confirmed superior performance of the curated gene set in both external datasets (*P* < 1 × 10^−64^; Extended Data Fig. 9a). Although FIB-4 was originally developed for the detection of advanced fibrosis, we include it here as a commonly used non-invasive clinical benchmark.

We further tested model generalizability by applying the transcriptomics-trained classifier to plasma proteomic data, where it maintained strong performance (AUC = 0.83; 0.79 for the 15-gene subset) significantly outperforming the published three-gene panel (DeLong test; *P* = 0.00006 and 0.05, respectively, for the 57 and 15-gene classifiers; Fig. 5e, Extended Data Fig. 9b and Methods). Together, these results demonstrate that the plasma-based gene signature generalizes across cohorts, outperforms established non-invasive tests and directly translates into blood proteomics.

To enable continuous patient positioning along the disease trajectory, we trained a random forest regression model using the 57-gene signature. In the UCAM/VCU cohort, the model achieved strong and consistent predictive performance (*R*^2^ = 87.9%, *P* = 1.66 × 10^−8^; mean squared error (MSE) = 0.008 across 1,000 iterations of fivefold cross-validation), accurately recapitulating increasing MASLD severity (Extended Data Fig. 9c,d). Validation in the independent EPoS cohort^8^ confirmed this performance (*R*^2^ = 85.1%, *P* = 8.04 × 10^−62^), with predicted positions tracking progressive MASLD stages (Extended Data Fig. 9e), whereas random 57-gene signatures did not (*R*^2^ = 0.14; Extended Data Fig. 9f).

Further validation in the Gubra dataset^25^ (Methods) demonstrated similar accuracy (*R*^2^ = 85.6%, *P* = 1.4 × 10^−26^), correctly separating healthy or obese individuals from patients with MASLD or MASH along the trajectory (Fig. 5f and Extended Data Fig. 9g), with 89.2% of early-trajectory individuals classified as healthy/obese and 89.7% of late-trajectory individuals as MASLD/MASH. Finally, applying the same model to plasma proteomics data successfully recapitulated disease progression at the protein level (*P* = 1.09 × 10^−18^; Fig. 5g,h). Together, these results demonstrate that the 57-gene signature robustly predicts MASLD progression across cohorts and omics layers, supporting its potential for non-invasive staging and longitudinal monitoring.

**Extended Data Fig. 10:**
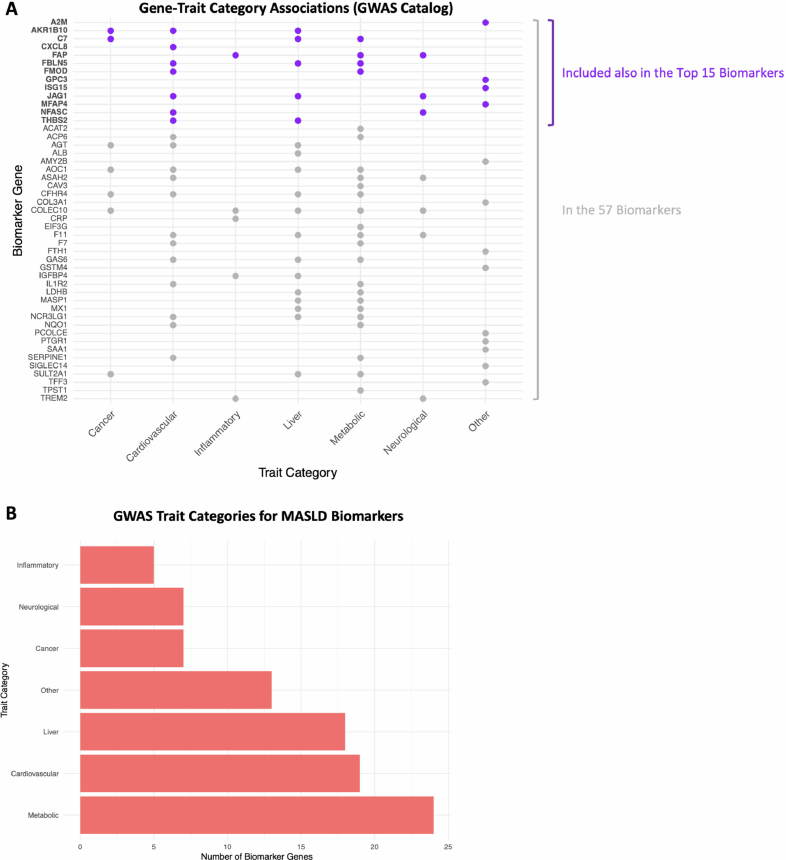
Genetic support for our 57-biomarker set. **A**. Gene-trait category associations based on the GWAS catalogue. Of those, 47/57 biomarkers were associated with at least one trait (Supplementary Table 16), while 13 biomarkers were part of our top 15 biomarker list (selected using the elbow method). **B**. Number of biomarkers associated with metabolic, cardiovascular, liver, cancer, neurological or inflammatory traits. Those associated with a different trait are classified as “other”. Source data

To assess genetic support for the 57-gene MASLD biomarker panel, we queried the GWAS Catalogue (v.1.0)^60^. A total of 47 of the 57 genes were associated with at least one complex trait, predominantly related to metabolic, cardiovascular and liver phenotypes (Extended Data Fig. 10a,b and Supplementary Table 16). Nearly half of the genes (27 of 57; 47.4%) were linked to multiple trait categories, suggesting pleiotropy. Enrichment analysis confirmed significant overrepresentation of metabolic (FDR = 2.33 × 10^−5^), cardiovascular (FDR = 0.0021) and liver-related traits (FDR = 0.0032) relative to the background (Fig. 5i). Although GWAS overlap alone does not establish causality, the enrichment of trajectory-associated genes near MASLD GWAS loci supports consistency with existing genetic studies, supporting the biological relevance of the biomarker panel.

To explore potential therapeutic intersections, we mapped approved and investigational compounds to the 57-gene biomarker panel (Supplementary Table 17). This revealed drug–target associations spanning core MASLD processes. For example, small molecules targeting COL3A1 have indications related to ECM remodelling and fibrotic disorders (for example Dupuytren contracture and Peyronie’s disease); those targeting CXCL9 and SERPINE1 are indicated for inflammatory and immune signalling-related conditions (for example, chronic bronchitis, Alzheimer’s disease and various cancers). Small molecules with indications for coagulation and vascular diseases, such as atrial fibrillation, acute coronary syndrome or chronic kidney disease, target F11 and AGT; metabolic or hepatocellular stress pathway-related indications were linked to ACAT2 and GPC3. Although exploratory, these patterns reinforce the biological relevance of the biomarker panel and illustrate how a trajectory- and network-informed framework can support hypothesis generation for biomarker-guided, stage-aware therapeutic prioritization in MASLD.

Together, these results define a network-anchored and genetically supported biomarker framework that captures MASLD progression as a continuous molecular trajectory and enables robust, non-invasive stratification of disease stage across cohorts and molecular layers.

## Discussion

MASLD has become a leading cause of chronic liver dysfunction and liver transplantation worldwide^2,61^, yet its clinical assessment and mechanistic interpretation still rely primarily on static, histology-defined stages. These semi-quantitative categories only partially capture the continuous and heterogeneous nature of disease progression and provide limited insight into the regulatory processes that govern transitions between states. Here, we present a data-driven framework that reconstructs a population-level molecular continuum of MASLD progression from cross-sectional liver transcriptomes. This trajectory is not a simulation or patient-level predictor, but a statistical representation of shared molecular states aligned with histological phenotypes across cohorts, offering a scalable alternative for studying disease progression when longitudinal biopsy data are limited or unattainable.

The inferred trajectory showed strong concordance with steatosis, ballooning, inflammation, fibrosis and NAS and identified a robust 145-gene signature predictive of histological scores that was reproducible across multiple independent datasets, including a large multicentre cohort and a longitudinal dataset with paired biopsies. Although MASLD progression is not strictly unidirectional at the individual level, the reproducibility of this axis across cohorts supports the existence of a dominant molecular progression pattern shared across patients. By design, our framework emphasizes common disease mechanisms rather than subgroup-specific modifiers, such as sex, diabetes status or body mass index, which were corrected for during integration or did not dominate trajectory placement in this setting. This focus highlights convergent regulatory programmes that may be broadly targetable across patient populations, while providing a foundation for future stratified analyses as larger, metadata-rich cohorts become available.

Integrating coexpression analysis with TF enrichment and upstream pathway information yielded a MASLD regulatory network enriched for established disease genes and validated in an independent large cohort. While module–phenotype associations remain correlative, anchoring the network to TFs and signalling pathways improves interpretability of upstream regulatory processes, as we have previously demonstrated^27^. TF-based pathway analysis refined established MASLD biology by showing that endoplasmic reticulum stress-mediated pathways (R-HSA-380994) and KEAP1–NFE2L2 oxidative-stress signalling (R-HSA-9755511) were selectively enriched in ballooning- and fibrosis-associated TF sets, rather than in steatosis-associated TF sets, suggesting that these stress-response programmes emerge primarily during hepatocyte injury. In addition, fibrosis-specific enrichment of TP53 regulatory signalling (R-HSA-3700989) and adherens junction interactions (HSA-418990) highlights transcriptional and structural remodelling processes that are less emphasized in conventional MASLD pathway analyses.

By resolving progression into overlapping SWs rather than discrete stages, the framework captured dynamic regulatory behaviours that traditional staging obscures. Immune-related TFs and pathways, including NF-κB and STAT signalling, were sustained across the trajectory, consistent with chronic inflammatory signalling in MASLD. In contrast, profibrotic and myogenic TFs, including MEF2 family members, exhibited a transient peak during mid-progression.

Pathway-level analysis further revealed temporally ordered activation of hypoxia responses, ECM remodelling and growth factor signalling (for example, WNT^62^, PDGF^63^ and TGF-β^64^), alongside non-monotonic metabolic changes. Persistent upregulation of glucose metabolism across the trajectory is consistent with hepatic and systemic insulin resistance. In contrast, lipid metabolism showed early suppression followed by partial reactivation, underscoring the complexity of metabolic dysfunction during disease progression.

MASLD progression is accompanied by shifts in liver cell composition driven by cellular plasticity, including stellate cell activation and hepatocyte–cholangiocyte transdifferentiation^65^. Cell-type deconvolution showed reduced hepatocyte signal and increased contributions from immune, cholangiocyte and fibroblast cell types, consistent with known histological changes. Macrophage signals increased along the trajectory and aligned with inflammatory pathways, supporting their established role in steatohepatitis^66^. Fibroblast-associated signatures, largely reflecting hepatic stellate cells within the reference atlas, were linked to ECM remodelling, whereas metabolic dysregulation was predominantly hepatocyte-driven. Although bulk deconvolution primarily supports relative trends rather than precise cell proportions, these findings are consistent with current models of MASLD and motivate future validation using single-cell^65^ and spatial^67^ approaches.

A key contribution of this study is the derivation of a 57-gene plasma-accessible biomarker panel, anchored in the MASLD regulatory network and validated across independent cohorts and omics layers. Unlike biomarker approaches based solely on predictive feature selection, this panel prioritizes mechanistic relevance by linking circulating markers to disease-associated regulatory programmes and to genes supported by MASLD GWAS associations, several of which, including *NFASC*, *NCR3LG1*, *EIF3G*, *TPST1*, *AMY2B* and *AOC1*, have not been widely studied in MASLD. The biomarker panel outperformed established non-invasive scores, including FIB-4, APRI and NFS, in distinguishing advanced fibrosis and enabled continuous placement of patients along the molecular trajectory. Furthermore, it aligns with established fibrosis-associated signatures, overlapping with clinically validated markers such as A2M (component of the NIS4 fibrosis test^68^), FAP (associated with PRO-C3 related fibrogenesis^69,70^) and THBS2 (refs. ^71,72^). Notably, our biomarker panel resolves into mechanistic axes central to MASLD progression, including ECM remodelling, immune activation, metabolic stress and coagulation–vascular pathways^73^.

Although these biomarkers are not yet ready for clinical application, their consistent performance across transcriptomic and proteomic datasets and their relevance to known processes underlying MASLD progression support the value of network-informed biomarker discovery in MASLD and motivate prospective validation. In this context, the observation that these biomarkers intersect with known pharmacological targets across fibrotic, inflammatory, vascular and metabolic pathways further highlights the potential of a trajectory-informed framework to support stage-aware therapeutic hypothesis generation.

This study has limitations. The trajectory captures shared molecular progression but cannot infer causality or predict individual disease courses. Cross-sectional data limit inference on reversibility and treatment effects, and subgroup-specific modifiers such as sex, diabetes and medication use are not explicitly modelled. Nonetheless, the resulting MASLD network provides a structured framework for contextualizing molecular dysregulation, identifying stage-associated regulatory programmes, and prioritizing candidate pathways for further investigation. Incorporation of longitudinal sampling, richer clinical metadata and single-cell or spatial data will be important next steps to refine this framework and extend it toward stratified and personalized analyses.

In summary, this work provides a unified, trajectory-based molecular framework for MASLD that organizes established disease mechanisms into a coherent and reproducible progression model. By integrating regulatory networks, pathway dynamics and non-invasive biomarkers, the framework advances beyond static staging to place patients along a continuous molecular progression axis. Beyond generating mechanistic hypotheses, this framework enables the identification of stage-specific regulatory programmes that may help prioritize candidate therapeutic targets and biomarkers for further investigation. In practice, this framework can be applied to (1) retrospective cohort data to map disease mechanisms; (2) biomarker-driven stratification of patients in clinical trials; and (3) longitudinal monitoring of disease progression or regression using plasma measurements. While developed here for MASLD, the approach is applicable to other progressive metabolic diseases characterized by cross-sectional molecular data.

## Methods

### Datasets

Two publicly available human datasets were used: the ‘UCAM’ dataset^24^ (58 consecutive patients recruited at the MASH Service, Cambridge University Hospital) and the VCU dataset^9^ (4 obese bariatric controls without MASLD and 74 patients spanning the MASLD spectrum). Raw transcriptomic data and associated metadata were downloaded from ArrayExpress (E-MTAB-9815) and NCBI Gene Expression Omnibus (GEO) (GSE130970), respectively. Dataset details are provided in Supplementary Tables 1 and 2. Sample_5 was excluded as an outlier (Supplementary Fig. 1a,b).

For trajectory inference validation, three publicly available transcriptomic datasets were used (Gubra^25,74^, EPoS^8^ and Fujiwara^26^) and the Govaere proteomics dataset^58^ was used for biomarker analysis. The Gubra dataset includes 26 healthy individuals (14 healthy weight and 12 overweight) and 31 patients with MASLD or MASH from Copenhagen University and Aarhus University Hospital. The EPoS dataset comprises 168 patients with MASLD or MASH from the multicentre European MASLD Registry^75,76^ and includes MASLD category, NAS, fibrosis stage and FIB-4 scores. The Fujiwara dataset^26^ contains longitudinal transcriptomic data from 58 patients with MASLD with paired liver biopsies spanning the disease spectrum. As non-invasive test scores were not provided, FIB-4 (ref. ^77^), APRI^78^ and NFS^79^ were calculated from available metadata using published definitions (Supplementary Table 18). The Govaere dataset^58^ includes paired plasma proteomics and liver transcriptomics from 191 patients, including samples overlapping with EPoS, enabling cross-omics validation and biomarker analysis. Datasets were selected based on the availability of liver transcriptomic data with detailed histological annotation and sufficient coverage across MASLD stages to enable model development and independent validation.

### RNA-seq analysis and data processing

Quality control of raw FASTQ files was performed using FastQC (v.0.11.9; http://www.bioinformatics.babraham.ac.uk/projects/fastqc). Reads were aligned to the human GRCh38 reference genome using hisat2 (v.2.1.0)^80^, and gene-level counts were obtained with HTSeq^81^ (v.0.11.1). Ensembl gene IDs were mapped to HUGO Gene Nomenclature Committee (HGNC) symbols using biomaRt (v.2.54.0)^82^.

Quantile normalization was applied across datasets, followed by batch-effect correction using COMBAT^83^ from the sva R package (v3.20.0), with sex included as a covariate (Supplementary Fig. 1a,b). Post-correction quality control showed consistent gene expression distributions within each dataset (Supplementary Fig. 1c,d). Correlation analyses across low (bottom third), medium (middle third) and high (top third) expression genes confirmed high concordance before and after correction (Supplementary Fig. 1d). The first principal component correlated with key MASLD features but not with sex (Supplementary Fig. 1e,f), and expression patterns of known MASLD-associated genes were preserved (Supplementary Fig. 1g), supporting the robustness of the correction.

### Discrete patient stratification for annotation of trajectory

Patients (*n* = 135) were stratified into mild (MASH F0), moderate (MASH F1–2) and severe (MASH F3–4) groups based on histological scoring using the CRN system^3^, following the previous NAFLD/NASH definitions. This stratification was used to aid interpretation, as described previously^24,76^ (Supplementary Table 1).

### Pseudo-temporal ordering of patients

Pseudo-temporal ordering was inferred using Slingshot^84^ (v.2.18.0). The resulting trajectory aligned with PC1, which correlated with MASLD features and explained 9% of the variance in the scaled and normalized data (~55% pre-normalization; Supplementary Fig. 1a,b), with no significant association with confounders such as age (Supplementary Fig. 1e).

This dimension captures a continuous molecular progression consistent with histological phenotypes and is independent of variables such as sex or T2DM, and was therefore interpreted as a pseudo-temporal MASLD trajectory (low values, early stages; high values,advanced^84^ fibrosis). For the independent datasets, the analysis was repeated using both the full transcriptome and the 145-gene signature derived from random forest classification (see below). Patient ordering along the trajectory is provided in Supplementary Table 1.

### Extraction of 145 gene signatures using random forest classification

Gene loadings from the rotation parameter of the prcomp function (R stats package) were used to rank genes by their contribution to variance along the disease trajectory. The top 200 genes were selected to train a random forest classifier (R package randomForest, v.4.6.14) to predict disease stage (MASL, MASH F0–1, MASH F2 and MASH F3–4) and histological features (steatosis, inflammation, ballooning and fibrosis). Model accuracy was assessed using fivefold cross-validation repeated 1,000 times. Genes with positive ‘mean decrease accuracy’ were retained, resulting in a 145-gene signature (Supplementary Table 1), further used to train a final model. Performance was evaluated across three independent cohorts (Gubra^25,74^, EPoS^8^ and Fujiwara^26^), demonstrating generalizability. AUC values for binary classifications are provided in Supplementary Table 3.

### Patients’ stratification into sliding windows

Ordered samples along the trajectory were partitioned into overlapping SWs to characterize molecular changes across pseudo-time. To define the optimal configuration, we developed a graph-based method evaluating all possible SW sequences given window sizes *S* = [*S*_*1*_*,…, S*_*m*_] and overlap percentage *α* between adjacent windows.

Samples were indexed by their ranking along the first principal component. Using an iterative process, a directed acyclic graph (DAG) was constructed where nodes represent groups of consecutive samples (candidate SWs) and directed edges define transitions between them. An empty starting node was first created as the origin of all paths in the DAG. In the first iteration, a set of nodes was generated using the input window sizes, representing candidates of SW_1_. Edges were created from the starting node to the SW1 nodes. In subsequent iterations, each existing node was extended forward along the index order, to generate candidates for the next SW with overlap *α*. This process continued until all samples were assigned, and terminal nodes were connected to an end node. All paths from start to end represent candidate SW sequences (Extended Data Fig. 3a).

Candidate paths were evaluated using differential expression analysis (DESeq2; ref. ^85^) between adjacent SWs. In each iteration, edges were retained if the number of differentially expressed genes (DEGs; FDR < 0.1) fell within *R* = [100, 1,000], reflecting sufficiently strong but not excessive differentiation. Paths containing edges outside this range were pruned. To maintain DAG connectivity, DEG thresholds were adaptively relaxed such that the lower bound did not exceed the 80th percentile and the upper bound did not fall below the 20th percentile of observed DEG counts.

Additional filtering reduced DAG complexity: (1) for each node, incoming edges were grouped by the index of the starting sample, retaining the edge with the highest DEG count (or largest span if tied); (2) edges below the lower DEG threshold were used to compute a median DEG value, and edges with fewer DEGs were removed while preserving DAG connectivity (Extended Data Fig. 3b).

Remaining paths were ranked using the product of three max-scaled criteria: median DEG count, number of edges above the lower DEG threshold and coefficient of variation of DEG counts along the path. Multiple configurations of SW sizes and overlap scores (([6,15], 0.2), ([8,17], 0.25), ([8,24], 0.3), ([10,20], 0.3) and ([12,25], 0.35)), were evaluated, and the optimal path was selected across all resulting graphs (Extended Data Fig. 3c and Supplementary Table 5).

### Identification of gene expression modules associated with MASLD histological phenotypes

Weighted gene coexpression network analysis (WGCNA) was performed as previously described^27^ using the R package WGCNA^28^ (v.1.73.0) on the full transcriptome to identify MASLD-related gene modules. A signed network was constructed using a soft-thresholding power of 4 to approximate scale-free topology, and modules were calculated based on the topological overlap matrix using dynamic tree cutting (cutreeDynamic, dynamicTreeCut v.1.63.1).

Module–phenotype associations were assessed by regressing module eigengenes against MASLD phenotypes, including inflammation and age as covariates. Inflammation was included to account for its dominant transcriptomic signal and its contribution to the NAS score, enabling identification of modules associated with steatosis, ballooning, fibrosis and NAS beyond global inflammatory effects. Age was included as a known determinant of disease progression.

Linear models (lm, step; R stats package) were fitted as:

$$\begin{array}{l}\mathrm{MASLD}\,\mathrm{phenotype} \sim \mathrm{intercept}+\mathrm{eigen}\,\mathrm{genes}\,\mathrm{of}\,\mathrm{modules}\\ +\mathrm{inflammation}+\mathrm{age}\end{array}\,\,\,\,$$

Backward stepwise selection using the Akaike information criterion retained modules with significant coefficients (adjusted *P* < 0.05; Benjamini–Hochberg correction). Model performance was evaluated using fourfold cross-validation (crossv_kfold, modelr v.0.1.11) and MSE. WGCNA parameters (deepSplit, minClusterSize) were optimized by fitting models across parameter combinations and selecting those minimizing average MSE across phenotypes, yielding deepSplit = 4 and minClusterSize = 180. Final models were built using these parameters.

For downstream analysis, module gene sets were filtered based on Pearson correlation with their eigengene: genes with non-positive correlation were removed from modules with <1,000 genes, while for larger modules a cutoff at the 20th percentile was applied (Supplementary Table 6).

### Differential expression and transcription factor activity analysis

Differential expression analysis was performed using DESeq2 (ref. ^85^) (v.1.46.0). TF activity was inferred with VIPER^86^ (v.1.40.0) using the CollecTRI database^87^ (accessed May 2024) and assessed by Student’s *t*-test (Supplementary Tables 8 and 12). *P* values were adjusted using the Benjamini–Hochberg method. For both discrete disease stage stratification and trajectory-based analysis, each stage or SW was compared with the preceding one.

### Construction of the global MASLD network

A global interaction network capturing molecular processes across MASLD stages was constructed by integrating signalling and transcriptional regulation derived from WGCNA modules, differential expression, and TF activity across SWs. Upstream (signalling) and downstream (transcriptional) subnetworks were generated using the Prize-Collecting Steiner Forest (PCSF) algorithm^88^ (R package PCSF, v.0.99.1) and merged into a global network.

Prize-carrying nodes were defined using two complementary approaches. First, for each MASLD phenotype and associated significant modules, TF enrichment was performed using CollecTRI^87^ and EnrichR^89^ TF-regulon databases (ENCODE and ChEA Consensus TFs from ChIP-X^90^, JASPAR^91,92^ and TF protein–protein interactions; accessed May 2024), followed by Reactome^29^ pathway enrichment (accessed May 2024) on the enriched TFs to identify upstream signalling pathways (Supplementary Tables 8 and 9). All enrichment analyses were restricted to genes detected in the transcriptomic data and were performed using Fisher’s exact test, followed by Benjamini–Hochberg correction for multiple testing (FDR < 0.05). Filtered module genes (downstream), enriched TFs and pathway genes (upstream) defined the prize nodes.

Second, for trajectory-based differential expression, selected TFs were deregulated in at least one SW (FDR < 0.05; Supplementary Table 12), their associated pathways were identified as above (Supplementary Table 9), and their regulons (CollecTRI^87^) were intersected with WGCNA modules. These regulon genes (downstream), deregulated TFs and pathway genes (upstream) defined the prize nodes for the deregulated network.

The reference interaction network was assembled from Reactome^29^, Kyoto Encyclopedia of Genes and Genomes^93^, HumanCyc^94^, ReconX^95^, provided by Pathway Commons^96^ (v.12) and OmniPath^97^ (OmniPathR v.3.8.0). Weakly supported OmniPath interactions were removed by retaining those above the 0.5 (resource count) and 0.75 (curation effort) quantiles. Dead-end metabolites and inhibitory edges were excluded, and protein complexes were expanded into fully connected subgraphs, yielding a network of 12,549 nodes and 205,706 edges (Supplementary Table 19).

PCSF was run on each of the five prize-node sets using a randomized variant (edge cost = 0.001, node prize = 1, ten iterations with added noise) to generate subnetworks, which were merged into a global network using igraph (v2.2.1). Only functionally annotated genes were retained. Edge weights were assigned based on Gene Ontology Biological Process semantic similarity^98^ using GOSemSim^99^ (v.2.29.2), averaged across Resnik^100^, Lin^101^ and Wang^102^ metrics, followed by Laplacian normalization to reduce hub bias^103^. The final MASLD network comprised 7,165 nodes and 69,314 edges (Supplementary Table 10).

Network relevance was assessed by enrichment analysis (using Fisher’s exact test) against two independent MASLD gene sets: a literature-curated signature (164 genes^73,104–108^; Supplementary Table 11) and the WikiPathways ‘NAFLD pathway’ from MSigDB^43^ (155 genes; 15-gene overlap), using Fisher’s exact test.

### Disease stage-specific network analysis

Deregulated components of the MASLD network were identified for each SW using network propagation implemented in igraph (v.2.2.1). The personalized PageRank algorithm^109^ was applied using node weights derived from differential expression (non-TFs) and TF activity analyses (TFs). Separate weight vectors were defined for up- and downregulation (${W}_{+}$ and ${W}_{-}$).

For node *i*, weights were assigned based on the sign of deregulation and the log-transformed adjusted *P* value (capped at 10):

- $$\begin{array}{l}{\mathrm{If}}\,{\mathrm{sign}}\,({\mathrm{deregulation}})=+1:\\{\mathrm{Weights}}_{i}\,:\{{W}_{+}=\min (-{\log }_{10}(P{\mathrm{adj}})+{1,10}),\,{W}_{-}=1\}\end{array}$$
- $$\begin{array}{l}{\mathrm{If}}\,{\mathrm{sign}}\,({\mathrm{deregulation}})=-1:\\ \,{\mathrm{Weights}}_{i}\,:\{{W}_{+}=1,{W}_{-}=\min \,(-{\log }_{10}(P{\mathrm{adj}})+1,\,10)\}\end{array}$$

Propagation was performed for each SW and direction. Statistical significance of PageRank scores was assessed using 1,000 permutations with randomized weights, and empirical *P* values were calculated as the fraction of random scores lower than the observed value.

The analysis was repeated for damping factors 0.5, 0.7 and 0.85, and genes with *P* value < 0.05 across all runs were considered significantly propagated. Subnetwork signatures were extracted as induced subgraphs after removing isolated nodes. Gene activation scores per SW were defined as the mean log-transformed *P* values across the three runs.

### Definition of disease-relevant pathways

Reactome^29^ enrichment analysis (Fisher’s exact test, Benjamini–Hochberg, FDR < 0.05; Supplementary Table 13) was used to link differentiated subnetworks in each SW to biological pathways, generating SW- and direction-specific enriched terms. To derive a unified, non-redundant set of MASLD-relevant pathways, a slim Reactome hierarchy was constructed and used as a reference framework.

Reactome parent–child relationships were encoded as a graph (igraph Python v.0.11.4). Information content (IC)^100^ and semantic value (SV)^110^ were computed from gene annotations to quantify terms specificity. Terms with IC and SV above the 25th percentile were removed as highly specific, reducing the graph from 2,655 to 315 terms. Semantic redundancy was further reduced by comparing child–parent IC and SV differences: child terms were removed if both differences were below the median and the parent was not directly connected to the root, yielding a slim graph of 225 terms (Supplementary Table 20).

MASLD-relevant pathways were defined using this framework. Reactome enrichment of the global MASLD network (FDR < 0.05) was intersected with the slim graph to generate a reference subgraph. SW-level enriched pathways (FDR < 0.05) were examined whether they were present in the reference subgraph or could be mapped to ancestral terms within it. If they failed to be mapped, they were excluded.

Pathways with <10 genes in SW subnetworks were removed as highly specific. This resulted in a MASLD-specific subgraph of 141 pathways, from which, 111 pathways were finally selected by retaining leaf terms and their parents, excluding those whose child terms covered >80% of their annotations (Extended Data Fig. 6 and Supplementary Tables 13 and 20).

Pathway activation scores were computed from gene-level activation scores (see ‘Disease stage-specific network analysis’). For each pathway, a subnetwork was defined by intersecting its annotation with the MASLD network. Activation scores per SW were calculated as the sum of gene scores weighted by PageRank centrality within the subnetwork. Cumulative scores were obtained by progressive summation across SWs (Extended Data Fig. 6 and Supplementary Table 13).

### Cell-type deconvolution analysis

The Liver Cell Atlas^57^ (10x single-cell RNA-seq from healthy human liver) was used as the reference. The dataset was re-analysed using standard single-cell workflows. Genes expressed in fewer than three cells and cells with fewer than 200 detected genes were excluded. Cells with >7,000 features (putative doublets) or >5% mitochondrial content were also removed. Deconvolution was performed using the CATD pipeline^111^ with raw counts as input (normalization and preprocessing performed internally). A subset of top-performing methods was used (EpiDISH^112^, bseqsc^113^, DWLS^114^, CIBERSORT^115^, FARDEEP^116^ and BayesPrism^117^), selected based on benchmarking across multiple metrics. Consensus estimates were computed as the mean cell-type proportion across methods (Supplementary Table 14).

### Identification of cell-type-specific deregulated pathways

To distinguish effects of cell-type composition from cell-intrinsic deregulation, we implemented the following workflow. Using SWs stratification and corresponding cell-type proportions, Gaussian kernel density functions were fitted for each cell type per SW. These were used to generate pseudo-bulk RNA-seq samples by sampling healthy cells from the single-cell reference dataset^57^. For each SW, the number of pseudo-samples (6–20) and library sizes (20–50 million counts) were randomly assigned. Differential expression, TF activity and network analyses were then performed using the same pipeline as for the real data, assuming these pseudo-signatures reflect variation driven solely by cell-type composition. A total of 30 pseudo-datasets were generated, and results were averaged across replicates.

Network analysis of pseudo-datasets identified deregulated components, which were compared with real SW subnetworks. Genes shared between real and pseudo signatures with lower activation in real data, were classified as composition-driven and excluded from MASLD-relevant pathways. Remaining genes were considered cell-intrinsic.

To assign pathways to specific liver cell types, extended marker sets were derived from the single-cell dataset using Seurat (v.5.3.1)^118^ and restricted to genes present in the MASLD network. Marker sets were expanded using the PCSF algorithm (same parameters as above; PCSF v.0.99.1) to include proximal network genes. Enrichment of deregulated pathways in cell-type marker sets was assessed (Fisher’s exact test, Benjamini–Hochberg, FDR < 0.05; Supplementary Table 15).

### Machine-learning models for non-invasive MASLD biomarker prediction

#### Random forest classification for fibrosis stage prediction

Govaere et al.^58^ identified 194 genes with correlated liver transcriptomic and plasma proteomic expression in 191 histologically characterized NAFLD cases (F0–F4; NASH-CRN scoring) from the European NAFLD Registry (ClinicalTrials.gov NCT04442334).

From this set, a curated panel of 57 genes was derived by intersecting with the differentiated component of the MASLD network. A random forest classifier was trained to distinguish early (F0–F2) from advanced fibrosis (F3–F4) using the UCAM/VCU cohort, with fivefold cross-validation repeated 1,000 times. Gene importance scores were used to select 15 genes via the elbow method, and a second classifier was trained on this subset.

Model performance was evaluated using receiver operating characteristic (ROC) analysis (AUC, sensitivity, specificity and accuracy). External validation was performed on the EPoS^8^ and Fujiwara^26^ datasets without retraining. Benchmarking included comparison with FIB-4, APRI, NFS (when available) and a published three-gene panel^59^ (*IGFBP7*, *SSC5D* and *SEMA4D*). Model robustness was assessed using 300 random 57-gene sets and comparing their AUC distributions with that of the curated panel.

To assess cross-omic translatability, the model was applied to plasma proteomics data^58^ using the same 57-gene (protein) signature, with protein abundances *z*-score normalized. Predictions were evaluated against fibrosis stage (F0–F2 versus F3–F4) using ROC analysis, with optimal thresholds determined by Youden’s J index. No feature selection or model training was performed on proteomics data; all model development was conducted exclusively on independent transcriptomic cohorts.

#### Random forest regression for trajectory position prediction

Random forest regression models were developed to predict patient position along the MASLD trajectory using the 57- and 15-gene signatures. For each model, data were split into training (80%) and testing (20%) sets, and performance was evaluated over 1,000 iterations using mean *R*^2^ and the Pearson correlation (and corresponding *P* value) between predicted and full-transcriptome-derived trajectory positions.

Expression data were *z*-score-normalized based on the 194-gene set identified by Govaere et al.^58^, enabling predictions with partial proteomic measurements and supporting potential translation to plasma-based inference.

Models trained on the UCAM/VCU cohort were applied without retraining to independent transcriptomic datasets (Gubra and EPoS) and to the Govaere plasma proteomics dataset^58^. Generalizability was assessed by comparing predicted and reference trajectory positions. As a control, 300 random 57-gene sets were evaluated per dataset, and predictive metrics were averaged to benchmark performance.

### GWAS catalogue mining and trait categorization

The GWAS catalogue^60^ (v.1.0) was used to extract associations between selected biomarkers and traits (Supplementary Table 16). For each gene, associated traits were grouped into predefined categories (metabolic, liver, cardiovascular, inflammatory, neurological, cancer and other) using regular expressions. The number of unique genes per category was calculated, and genes mapping to multiple categories were recorded. Gene–trait relationships were visualized using bar and dot plots.

Enrichment of trait categories among the 57 MASLD biomarkers relative to all GWAS Catalogue genes was assessed using Fisher’s exact test, with Benjamini–Hochberg correction applied (FDR < 0.05).

### Mapping of small molecules onto biomarker panel

Potential small molecules targeting the biomarker panel were identified using the ChEMBL database^119^. Biomarkers were classified as up- or downregulated across SWs, and compounds were selected based on their ability to counteract these changes (activators/agonists for downregulated targets; inhibitors/antagonists for upregulated targets). Drug–target mappings were compiled for key molecular events across trajectory stages (Supplementary Table 17).

### Reporting summary

Further information on research design is available in the Nature Portfolio Reporting Summary linked to this article.

## Supplementary information

Supplementary Information Supplementary Fig. 1 and Legend.

Reporting Summary

Peer Review File

Supplementary Data 1 Supplementary Data. Source data for supplementary figures. Sheet_A&B: PC1 and PC2 components of the UCAM/VCU normalized expression data before and after batch-effect correction, respectively. Sheet_C&D: Normalized counts before and after batch-effect correction for the different stages of MASLD in SANYAL (VCU) and UCAM human datasets, respectively. Sheet_E&F: Correlations of the MASLD-related and non-related variables with the first ten principal components. Sheet_G: Normalized (before and after batch correction) expression data for genes previously linked to MASLD. Overall, these data are used to produce the plots in Supplementary Fig. 1, validating the quality of the datasets used in our analysis.

Supplementary Tables Supplementary Tables 1–20.

## Source data

Source Data Fig. 1 Data showing the position of the patients along the disease trajectory, for the main and validation human datasets, as well as the Kyoto Encyclopedia of Genes and Genomes pathways that are enriched in the top 145 genes derived from our analysis.

Source Data Fig. 2 Data derived from multiple computational analyses used to construct the global MASLD network. They summarize the associations between the calculated gene coexpression modules and MASLD-related phenotypic variables derived from linear modelling, their dynamic expression patterns along the disease trajectory, their regulatory links to transcription factors calculated by enrichment analysis on multiple TF-regulon databases, and the enrichment of the resulting MASLD-related networks in previously defined MASLD gene sets.

Source Data Fig. 3 Data derived from TF activity analyses based on the SW patient stratification, as well as two discrete stratifications defined by steatosis, activity and fibrosis (SAF) and NAS scores. At each stage or SW, the examined group of patients was compared with the preceding one to calculate differential TF activities.

Source Data Fig. 4 Data illustrate the dynamic changes in cell type proportions along the disease trajectory (for specific cell types and categories of haematopoietic cells), inferred from cell type deconvolution of transcriptomic data. They also show the associations between cell-type-specific marker gene sets and pathways deregulated across the disease trajectory.

Source Data Fig. 5 Data derived from the random forest classification and regression models, for the Fujiwara and EPoS transcriptomic (external validation) datasets, the Govaere plasma proteomics dataset, the real versus predicted trajectory positions of the patients on the external Gubra dataset, the predicted trajectory positions of the patients according to the Govaere plasma proteomics dataset (using the whole proteomics dataset or only the 57 Biomarker signature), and the enrichment of MASLD biomarkers in specific trait categories.

Source Data Extended Data Fig. 1 Positions of the patients on the disease trajectory (pseudotimes; x_points), and colours used for each patient according to the NAS, steatosis, ballooning, inflammation and fibrosis scores, as well as symbols (pch) and size (cex) used to produce the respective plots, and additional statistics describing Extended Data Fig. 1c.

Source Data Extended Data Fig. 2 Data showing the trajectory positions (pseudotimes) of the patients for the external validation (EPoS and GUBRA) datasets. The data supporting the Fujiwara longitudinal dataset show the shift in the patients’ trajectory positions between the two biopsies.

Source Data Extended Data Fig. 4 Data showing the association, represented as a one-hot encoded matrix, between the 57 TFs commonly identified as deregulated across SW-, SAF- and NAS-based patient stratifications and enriched Reactome pathways.

Source Data Extended Data Fig. 5 Data derived from the network analysis in the UCAM/VCU and EPoS datasets for the SW-based patient stratification. The data illustrate Pearson correlation coefficients of cumulative TF activation scores, both at the individual TF level and across each SW.

Source Data Extended Data Fig. 6 Data derived from network analysis and the subsequent mapping of extracted network signatures to Reactome pathways, showing cumulative pathway activation scores along the disease trajectory. Pathways were selected based on enrichment analysis of the extracted network signatures, their intersection with enrichment results from the global MASLD network, and subsequent filtering of enriched terms using a pruned version of the Reactome graph. The activation scores were calculated by summing the activation scores of the genes associated with each pathway along the disease trajectory.

Source Data Extended Data Fig. 7 Data derived from the network analysis and the subsequent translation of extracted network signatures to Reactome pathways in the UCAM/VCU and EPoS datasets for the SW-based patient stratification. The data illustrate Pearson correlation coefficients of cumulative pathway activation scores, both at the individual pathway level and across each SW.

Source Data Extended Data Fig. 8 Additional data for Fig. 4a showing changes in cell type proportions for individually identified cell types, rather than grouping most immune-related cell types into broader categories.

Source Data Extended Data Fig. 9 Additional data to Fig. 5, from the random forest models for random gene signatures (transcriptomic data), sensitivity versus specificity optimal threshold selection (proteomics data), and real versus predicted patients’ trajectory positions

Source Data Extended Data Fig. 10 Processed data providing genetic-related information for the final 57 Biomarkers, including gene names, category (for example, metabolic, liver and cancer), and further details if they also belong to the top 15 selected biomarker signature.

## Data Availability

All data used in this study are publicly available as described in the Methods. The raw transcriptomic data and relevant metadata for the UCAM and VCU datasets were downloaded from Array Express (E-MTAB-9815) and NCBI’s GEO (GSE130970), respectively. The Gubra, EPoS and Fujiwara datasets were downloaded from NCBI’s GEO (GSE126848, GSE135251 and GSE193084, respectively). The Govaere proteomics data were downloaded from the supplementary files of the original publication^58^. Source data are provided with this paper.

## Code availability

The code used for the analyses, together with processed data required for reproduction, is available at https://github.com/kamzolas/MASLD–Continuous-trajectory-approach. This repository is also archived on Zenodo at 10.5281/zenodo.19816412 (ref. ^120^) to ensure long-term reproducibility and version control.
