# Supplementary material for: A data-driven framework reconstructs the molecular continuum of human MASLD progression
Source: Nat Metab. 2026 Jul 14;8(7):1545–62. doi: 10.1038/s42255-026-01543-7 (PMC13400313; doi:10.1038/s42255-026-01543-7)
Supplement: Supplementary file 1 — Supplementary Fig. 1 and Legend. [file 42255_2026_1543_MOESM1_ESM.pdf]

nature metabolism

Supplementary information https://doi.org/10.1038/s42255-026-01543-7

A data-driven framework reconstructs the molecular continuum of human MASLD progression

In the format provided by the authors and unedited

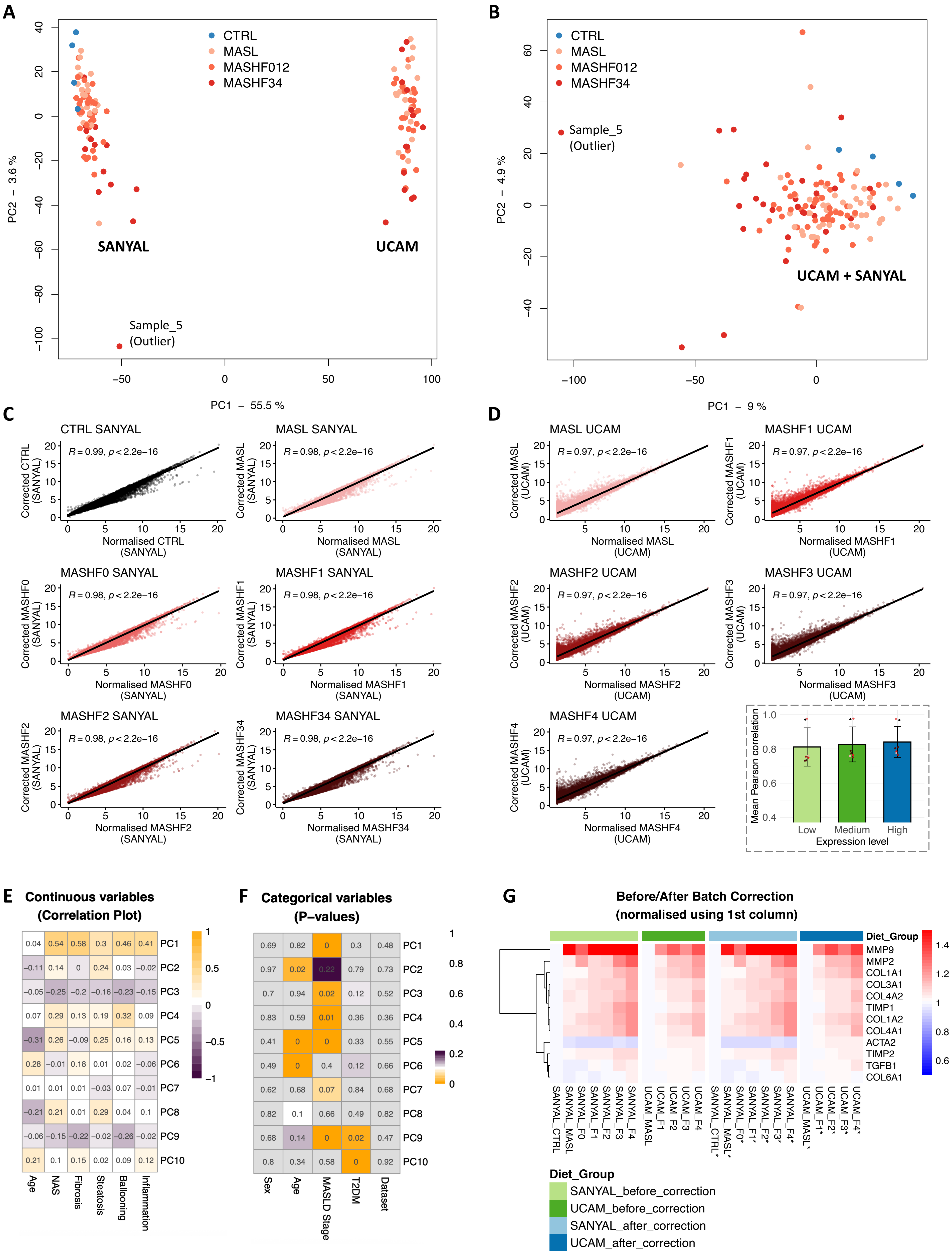
A
CTRL
MASL
MASHF012
MASHF34
SANYAL
UCAM
Sample_5
(Outlier)
PC2 – 3.6 %
PC1 – 55.5 %
B
UCAM + SANYAL
PC2 – 4.9 %
PC1 – 9 %
C
CTRL SANYAL
MASL SANYAL
MASHF0 SANYAL
MASHF1 SANYAL
MASHF2 SANYAL
MASHF34 SANYAL
R = 0.99, p < 2.2e−16
R = 0.98, p < 2.2e−16
Corrected CTRL (SANYAL)
Normalised CTRL (SANYAL)
D
MASL UCAM
MASHF1 UCAM
MASHF2 UCAM
MASHF3 UCAM
MASHF4 UCAM
R = 0.97, p < 2.2e−16
Mean Pearson correlation
Low
Medium
High
Expression level
E
Continuous variables
(Correlation Plot)
Age
NAS
Fibrosis
Steatosis
Ballooning
Inflammation
F
Categorical variables
(P–values)
Sex
Age
MASLD Stage
T2DM
Dataset
G
Before/After Batch Correction
(normalised using 1st column)
Diet_Group
MMP9
MMP2
COL1A1
COL3A1
COL4A2
TIMP1
COL1A2
COL4A1
ACTA2
TIMP2
TGFB1
COL6A1
SANYAL_before_correction
UCAM_before_correction
SANYAL_after_correction
UCAM_after_correction

Supplementary Figure 1. Quality control and validation for dataset integration. **A.** PCA plot of the merged UCAM/VCU quantile normalised counts. There is an apparent batch effect separating the patients into two clusters stemming from the dataset from which they are derived. **B.** COMBAT batch effect correction solved the problem of the batch effect with patients altogether in a cluster and a clear disease progression pattern from the right towards the later disease stages on the left. The outlier (sample 5) was removed from all downstream analyses. **C-D.** Normalised counts before and after batch effect correction, separately for each disease stage. The data suggest that COMBAT has not significantly altered gene expression, as indicated by the high correlation ($R > 0.97$) across all MASLD stages in the VCU (C) and UCAM (D) datasets. Inset in panel D shows the mean correlation in expression before and after correction stratified by low, medium, and high expression levels. The categories were defined by ranking genes by normalised expression in each stage and dividing them into three groups: High (top third), Medium (middle third), and Low (bottom third). The dots inside each bar correspond to the correlations of expression separately for each disease stage (dot colours in agreement with disease stage colours from panel C). **E.** Correlation of the principal components (PCs) with the variables of interest. The first 10 PCs have been correlated using Pearson correlation with 6 continuous variables of interest: age, NAS score, Fibrosis, Steatosis, Ballooning, and Inflammation. **F.** Heatmap of the quantile normalised counts before and after the merging/batch effect correction for UCAM and VCU datasets. The rows show genes previously linked to MASLD[120–123]. The data are shown in a "fold change vs the first disease stage" of each dataset, respectively ("red" = increase; "blue" = decrease; "white" = unchanged; "green datasets": UCAM and VCU before correction; "blue datasets": UCAM and VCU after correction; the asterisks "*" in the column names indicate that the samples have been batch-effect corrected). Note that the control group (the first column in each block) in the VCU dataset is labelled as CTRL, whereas in the UCAM it is labelled as MASL.
