# Supplementary material for: A data-driven framework reconstructs the molecular continuum of human MASLD progression
Source: Nat Metab. 2026 Jul 14;8(7):1545–62. doi: 10.1038/s42255-026-01543-7 (PMC13400313; doi:10.1038/s42255-026-01543-7)
Supplement: Supplementary file 2 — Reporting Summary [file 42255_2026_1543_MOESM2_ESM.pdf]

nature portfolio

Corresponding author(s): Antonio Vidal-Puig, Evangelia Petsalaki

Last updated by author(s): Apr 30, 2026

Reporting Summary

Nature Portfolio wishes to improve the reproducibility of the work that we publish. This form provides structure for consistency and transparency in reporting. For further information on Nature Portfolio policies, see our Editorial Policies and the Editorial Policy Checklist.

Statistics

For all statistical analyses, confirm that the following items are present in the figure legend, table legend, main text, or Methods section.

n/a	Confirmed	
☐	☒	The exact sample size (*n*) for each experimental group/condition, given as a discrete number and unit of measurement
☐	☒	A statement on whether measurements were taken from distinct samples or whether the same sample was measured repeatedly
☐	☒	The statistical test(s) used AND whether they are one- or two-sided<br>*Only common tests should be described solely by name; describe more complex techniques in the Methods section.*
☐	☒	A description of all covariates tested
☐	☒	A description of any assumptions or corrections, such as tests of normality and adjustment for multiple comparisons
☐	☒	A full description of the statistical parameters including central tendency (e.g. means) or other basic estimates (e.g. regression coefficient) AND variation (e.g. standard deviation) or associated estimates of uncertainty (e.g. confidence intervals)
☐	☒	For null hypothesis testing, the test statistic (e.g. *F*, *t*, *r*) with confidence intervals, effect sizes, degrees of freedom and *P* value noted<br>*Give P values as exact values whenever suitable.*
☒	☐	For Bayesian analysis, information on the choice of priors and Markov chain Monte Carlo settings
☒	☐	For hierarchical and complex designs, identification of the appropriate level for tests and full reporting of outcomes
☐	☒	Estimates of effect sizes (e.g. Cohen's *d*, Pearson's *r*), indicating how they were calculated

Our web collection on statistics for biologists contains articles on many of the points above.

Software and code

Policy information about availability of computer code

Data collection: Microsoft Excel – Microsoft Office 2019

Data analysis:

All analyses were performed using R (v4.4.1) and Python (v3.12.3) programming languages.
For NGS data analysis, FastQC (v0.11.9; https://github.com/s-andrews/FastQC; per-sample quality control), Hisat2 (v2.1.0; alignment to the GRCh38 reference genome), HTSeq (v0.11.1; gene-level quantification), and biomaRt (v2.54.0; gene annotation) were used.
For the human clinical feature characterisation, we used ANOVA (base R package; for the continuous variables) and chi-squared test (for the categorical variables).
For integrative analyses, including principal component analysis (PCA) and trajectory inference, the UCAM/VCU datasets were normalised using quantile normalisation (qnorm; standard R function) and integrated using COMBAT (sva package, v3.38.0), with sex included as a covariate. The function prcomp was used for the estimation of the principal components in the pca plots (package stats - v4.0.3). Patient pseudotemporal ordering was inferred using Slingshot (v3.22). Patients were then stratified along the trajectory into optimised overlapping sliding windows to enable stage-to-stage differential expression and network analysis.

1. The graph-based optimisation framework to stratify the patients into overlapping groups (sliding windows) was implemented using igraph (R package v.2.2.1).
2. Weighted gene co-expression network analysis (WGCNA; v1.73.0) was performed on the batch-corrected RNA-seq dataset to identify gene co-expression modules. The soft-thresholding power was selected to approximate scale-free topology in the resulting correlation network.
3. Linear regression (base R stats package) with cross-validation (modelr package v0.1.11) was used to assess module–phenotype associations, including inflammation and age as covariates. This analysis was repeated across multiple values of the deepSplit and minClusterSize WGCNA parameters to identify the set of modules that best predicted the MASLD phenotypes.
4. Differential expression analysis between pairs of adjacent sliding windows was performed using DESeq2 (v1.46.0), followed by adjustment

using the Benjamini-Hochberg method (FDR calculation). VIPER (v1.40.0) was used for transcription factor activity analysis with regulons retrieved from the CollecTRI database (accessed using the decoupleR package v.2.12.0), followed by statistical testing and FDR correction.
5. Transcription factor and pathway enrichment analyses were performed to associate module gene sets with transcription factors (based on the CollecTRI database and regulon databases retrieved from the EnrichR web application - May 2024) and, in turn, link those transcription factors to pathways (using the Reactome database; accessed May 2024). The statistical tests were performed using the fisher.test and p.adjust functions (package stats; v4.0.3).
6. The reference PPI network was constructed by integrating signaling and metabolic interaction databases from Pathway Commons (v12) and OmniPath, using OmniPathR (v3.8.0) and igraph (R package v2.2.1). The global MASLD network was derived as a subset of this reference network using the Prize-Collecting Steiner Forest (PCSF) algorithm (PCSF R package v0.99.1) together with igraph. GOSemSim (v2.26.0) and GO.db (v3.20.0) were used to score the edges of the MASLD network based on the functional similarity of interacting gene pairs.
7. Network analysis was performed using the PageRank algorithm of the igraph package (v.2.2.1). Statistical significance was estimated using randomised node weights (empirical test).
8. Pathway enrichment analysis of the extracted network signatures was performed using Reactome (accessed May 2024), applying Fisher's exact test with Benjamini–Hochberg correction. A pruned version of the Reactome graph was constructed to project the results along the MASLD trajectory using a concise set of pathways, implemented with igraph (Python package v0.11.4).
9. The reference single-cell data were processed using Seurat (v.5.3.1). Cell type deconvolution was performed using the CATD pipeline. The enrichment analysis used to identify disease-relevant cell types and pathways was applied using Fisher's exact test (scipy.stats Python package v.1..1.4) with Benjamini–Hochberg correction (statsmodels.stats.multitest Python package v.0.14.1).
10. The chembl_webresource_client Python library (v0.10.9 - used in Python 3.8 environment) was used to retrieve ChEMBL data for the biomarkers panel.

For the Machine Learning analysis part, we used the randomForest R package (v4.6.14). The Random Forest (RF) classifiers and regressors were trained exclusively on the internal UCAM/VCU dataset, using repeated 5-fold cross-validation. External validation was applied to the independent transcriptomic datasets (EPoS, Fujiwara, GUBRA) without re-training. For cross-omics assessment, trained models were applied to external plasma proteomics data using z-score-scaled protein abundance. Importantly, proteomics data were used exclusively for translatability purposes and not for feature selection or model training.
GWAS Catalog was used to map biomarkers to disease-associated traits, with enrichment assessed with Fisher's exact test followed by FDR correction.

Data visualisation was performed using the ggplot2 R library (v4.0.1) and the matplotlib (v3.6.3) and seaborn (v0.13.2) Python libraries.

All custom scripts, workflows and software requirements are publicly available on GitHub (https://github.com/kamzolas/MASLD---Continuous-trajectory-approach)

For manuscripts utilizing custom algorithms or software that are central to the research but not yet described in published literature, software must be made available to editors and reviewers. We strongly encourage code deposition in a community repository (e.g. GitHub). See the Nature Portfolio guidelines for submitting code & software for further information.

Data

Policy information about availability of data

All manuscripts must include a data availability statement. This statement should provide the following information, where applicable:

- Accession codes, unique identifiers, or web links for publicly available datasets
- A description of any restrictions on data availability
- For clinical datasets or third party data, please ensure that the statement adheres to our policy

No new patient data were generated as part of this study, and all data used are already publicly available. There are no restrictions on data access beyond those imposed by the authors in the original studies.
The UCAM dataset is available through the ArrayExpress platform (accession number: E-MTAB-9815) and the VCU dataset through GEO (accession number: GSE130970).
External transcriptomic datasets used for validation were retrieved from the GEO database using the following accession numbers: GSE126848 (GUBRA dataset), GSE135251 (EPoS dataset), GSE193084 (Fujiwara longitudinal dataset).
The Govaere plasma proteomics dataset is also publicly available, and the material used in this study was accessed through the supplementary material of the original publication (PMID: 37037945).
The processed data used to create the figures in the manuscript are available through the accompanying code on GitHub (https://github.com/kamzolas/MASLD---Continuous-trajectory-approach).
This repository is also archived on Zenodo for long-term reproducibility: https://doi.org/10.5281/zenodo.19816413 (This corresponds to GitHub release v1.0)

Research involving human participants, their data, or biological material

Policy information about studies with human participants or human data. See also policy information about sex, gender (identity/presentation), and sexual orientation and race, ethnicity and racism.

Reporting on sex and gender	This study has used already published human datasets. No new human data were generated or clinical data collected as part of this study.<br>Sex information was available for all analysed datasets and was incorporated as a biological covariate in integrative analyses where appropriate (e.g., batch correction). Gender identity information was not reported in the original datasets.
Reporting on race, ethnicity, or other socially relevant groupings	Information on socially relevant characteristics, including race and ethnicity, was not consistently available across the original studies and was not included in the analyses. Our study did not interpret results based on these variables.

Population characteristics	Population characteristics, including MASLD and fibrosis stage, and relevant clinical variables, were defined in the original studies and are summarised in the corresponding publications (and their associated metadata). Additionally, they are described in the relevant sections of this study, including the method and supplementary information parts.
Recruitment	No participants have been recruited for this study. All data were obtained from previous studies where specific criteria were followed as described in the original publications.
Ethics oversight	This analysis did not require additional ethics approval. All ethical approval requirements were followed in the original studies for the collection and use of human samples and associated clinical data.

Note that full information on the approval of the study protocol must also be provided in the manuscript.

Field-specific reporting

Please select the one below that is the best fit for your research. If you are not sure, read the appropriate sections before making your selection.

☒ Life sciences ☐ Behavioural & social sciences ☐ Ecological, evolutionary & environmental sciences

For a reference copy of the document with all sections, see nature.com/documents/nr-reporting-summary-flat.pdf

Life sciences study design

All studies must disclose on these points even when the disclosure is negative.

Sample size	Sample sizes have been mentioned in any relevant statistical analysis parts of our study. Given the retrospective nature of the study, statistical analyses were performed based on the human data availability following previously published datasets. Additional sample size details are reported in the corresponding publications and metadata.
Data exclusions	Data exclusions were limited to those required for quality control or missing information. Specifically, samples appearing as technical outliers or lacking essential metadata where excluded from the relevant parts of the analysis. No data were excluded based on study outcomes.
Replication	All analyses were generalised across multiple independent cohorts to assess robustness. Key findings were evaluated using external validation datasets and, where available, longitudinal data. All computational analyses are reproducible using the same code and input data found on GitHub (https://github.com/kamzolas/MASLD---Continuous-trajectory-approach).
Randomization	This study did not involve experimental interventions or enrollment of new patient participants. Thus, randomisation was not applicable, and where random sampling was used for computational purposes (e.g,. permutation testing), appropriate randomisation was implemented programmatically.
Blinding	Blinding was not available as this study used already published human datasets.

Reporting for specific materials, systems and methods

We require information from authors about some types of materials, experimental systems and methods used in many studies. Here, indicate whether each material, system or method listed is relevant to your study. If you are not sure if a list item applies to your research, read the appropriate section before selecting a response.

Materials & experimental systems

n/a	Involved in the study
☒	☐ Antibodies
☒	☐ Eukaryotic cell lines
☒	☐ Palaeontology and archaeology
☒	☐ Animals and other organisms
☒	☐ Clinical data
☒	☐ Dual use research of concern
☒	☐ Plants

Methods

n/a	Involved in the study
☒	☐ ChIP-seq
☒	☐ Flow cytometry
☒	☐ MRI-based neuroimaging

Plants

Seed stocks	n/a
Novel plant genotypes	n/a
Authentication	n/a
