# Supplementary material for: A data-driven framework reconstructs the molecular continuum of human MASLD progression
Source: Nat Metab. 2026 Jul 14;8(7):1545–62. doi: 10.1038/s42255-026-01543-7 (PMC13400313; doi:10.1038/s42255-026-01543-7)
Supplement: Supplementary file 3 — Peer Review File [file 42255_2026_1543_MOESM3_ESM.pdf]

nature portfolio

Peer Review File

A data-driven framework reconstructs the molecular continuum of human MASLD progression

Corresponding Author: Dr Evangelia Petsalaki

This file contains all reviewer reports in order by version, followed by all author rebuttals in order by version.

Version 0:

Reviewer comments:

Reviewer #1

(Remarks to the Author)
I have reviewed the earlier version of the manuscript. The manuscript has been substantially revised and improved. The inclusion of paired longitudinal samples provides useful supportive evidence that trajectory shifts are consistent with changes in NAS and fibrosis, although it does not fully establish that the trajectory represents true disease progression, as it is still largely derived from cross-sectional data. The authors appropriately acknowledge this limitation. Several points require further clarification.

1) Many of the molecular features highlighted, including inflammatory and fibrogenic pathways and the proposed 57-feature panel, have been widely reported in MASLD. To better demonstrate the added value of this study, a clearer discussion of the novel biological insights and newly implicated genes from this analysis would strengthen the manuscript.

2) Inconsistency in Figure 2 requires clarification. Some modules described as positively associated with disease severity in Figure 2B appear to decrease along the pseudo-temporal axis in Figure 2C. The relationship between module–phenotype correlations and their trajectory patterns should be explained to help readers interpret these results.

3) While the authors attempt to distinguish cell-intrinsic regulatory changes from shifts in cellular composition, the current analyses do not fully address whether the inferred pseudo-temporal trajectory itself is primarily driven by changes in cell-type proportions. Although genes whose expression could be explained by cell composition were removed in downstream analyses, it remains unclear whether the trajectory (e.g., PC1 or the ordering framework) is largely defined by variation in immune infiltration or other compositional shifts. To strengthen the claim that the trajectory reflects true regulatory reprogramming rather than cellular proportion changes, the authors should explicitly assess the contribution of cell-type composition to the principal axis underlying the pseudo-temporal ordering. For example, examining correlations between the trajectory axis and cell-type fractions, performing variance partitioning, or reconstructing the trajectory after regressing out cell-type proportions would help clarify whether the observed progression is independent of compositional effects.

(Remarks on code availability)

Reviewer #2

(Remarks to the Author)
The authors have addressed most of the previous concerns, and the innovative application of established computational methods has yielded numerous potentially interesting leads that may help deepen our understanding of disease progression mechanisms along the trajectory. However, the study appears better suited for hypothesis generation and motivating follow-up studies than for delivering clear actionable or translational insights at this stage.

Several issues remain that require clarification or more cautious interpretation. In Figure 5B, the claim that the proposed model outperforms established clinical scores (FIB-4, APRI, and NFS) based on ROC-AUC is not supported by the data shown, as FIB-4 appears to perform better. This discrepancy should be clarified. Moreover, the proposed model demonstrates lower sensitivity than existing clinical tests. Given that sensitivity is often more clinically relevant than

specificity for detecting advanced fibrosis, this limitation and its clinical implications should be discussed. Claims of “outperformance” should be supported by appropriate statistical evidence, including confidence intervals and formal tests comparing AUCs.

In Figure 4A, the interpretation that endothelial cell proportions remain relatively stable across the disease progression trajectory is not fully convincing. The figure instead suggests a clear upward trend, with endothelial cells increasing from approximately 15% to 30% at later stages. This pattern supports alternative biological interpretations, such as proliferation of liver sinusoidal endothelial cells or expansion of dysfunctional endothelial populations, which warrant discussion.

Finally, the use of the term “genetic evidence” is not sufficiently justified. Establishing genetic evidence for causality generally requires analyses such as colocalization and Mendelian randomization using appropriate instrumental variables. Mapping to GWAS signals alone is insufficient to support causal claims and should therefore be described more cautiously.

(Remarks on code availability)
The authors have added the code I previously requested. I have not tried to run the code myself.

Reviewer #3

(Remarks to the Author)
The article "Decoding MASLD Progression: A Molecular Trajectory-Based Framework for Modelling Disease Dynamics" by Kamzolas et al applies a biostatistical modeling approach to categorizing stages of disease in MASLD. The authors employ a number of techniques using available transcriptomic, proteomic, phenotypic, and histologic datasets to ascertain a molecular disease signature across the MASLD continuum. While the methodology, hypothesis, and clinical applications are of interest to the general reader and to liver disease specialists, the article lacks basic cellular biology techniques that align with the journal. Minor comments include a need to reduce the number of supplemental figures and cohere the critical pieces of the data.

Minor comments:

There is a need to reduce the number of supplemental figures and cohere the critical pieces of the data.
It is unclear why the authors chose the specific datasets they did (there are numerous available... what was the rationale for use of the ones chosen to build this model?)
It is unclear why the authors chose the putative signature genes a priori (for eg, what is the rationale for choosing the candidate biomarkers in Fig 5A?)
How relevant is it that this gene-based biomarker that applies across the MASLD continuum outperforms FIB-4, which was only established to predict fibrosis?
Were there any novel pathways found besides inflammation and fibrosis? Both of whose signatures increase along the histologic disease continuum?

(Remarks on code availability)

Reviewer #4

(Remarks to the Author)
I thank the authors for addressing my comments. The manuscript reads well and is defensible.

My only final question/comment is that the shift from histological modeling of MASLD progression to transcriptomic modeling seems like a major advance. This is not my area of expertise, so I did some quick searches to see if these ideas have been explored before. These papers seem to at minimum lay the groundwork for the current study:

Characterization of transcriptional modules related to fibrosing-NAFLD progression
Yi Lou, Guo-Yan Tian, Yu Song, Yin-Lan Liu, Yi-Dan Chen, Jun-Ping Shi & Jin Yang

Development of a novel non-invasive biomarker panel for hepatic fibrosis in MASLD
Lars Verschuren et al

Hepatic gene expression profiles differentiate presymptomatic patients with mild versus severe nonalcoholic fatty liver disease
Cynthia A Moylan et al

Human hepatic gene expression signature of non-alcoholic fatty liver disease progression, a meta-analysis
Maria Ryaboshapkina & Mårten Hammar

Pseudotime Analysis Imputes the Missing Liver NAFLD Status in Public RNA-Seq Cohorts

Tongyang Wang, Xiangmei Dou

I couldn't find citations for these in the manuscript. My request is whether these (and other cited studies that have done transcriptomic analysis and/or temporal analysis of MASLD) could be cited in the introduction or conclusion, to more clearly contextualize what the current study adds on top of them. Or, if these studies are not relevant, that would be fine to address as well.

(Remarks on code availability)

Version 1:

Reviewer comments:

Reviewer #1

(Remarks to the Author)
I went through the response letter and the latest version of the manuscript and I suggest the publication of the paper in its current form.

(Remarks on code availability)

Reviewer #2

(Remarks to the Author)
The authors have addressed all my previous concerns.

(Remarks on code availability)

Open Access This Peer Review File is licensed under a Creative Commons Attribution 4.0 International License, which permits use, sharing, adaptation, distribution and reproduction in any medium or format, as long as you give appropriate credit to the original author(s) and the source, provide a link to the Creative Commons license, and indicate if changes were made.
In cases where reviewers are anonymous, credit should be given to 'Anonymous Referee' and the source.
The images or other third party material in this Peer Review File are included in the article’s Creative Commons license, unless indicated otherwise in a credit line to the material. If material is not included in the article’s Creative Commons license and your intended use is not permitted by statutory regulation or exceeds the permitted use, you will need to obtain permission directly from the copyright holder.
To view a copy of this license, visit https://creativecommons.org/licenses/by/4.0/

We thank the reviewers and editor for their consideration and thoughtful comments. Below we detail our point-by-point response to them.

Reviewers comments:

Reviewer #1 (Remarks to the Author):

I have reviewed the earlier version of the manuscript. The manuscript has been substantially revised and improved. The inclusion of paired longitudinal samples provides useful supportive evidence that trajectory shifts are consistent with changes in NAS and fibrosis, although it does not fully establish that the trajectory represents true disease progression, as it is still largely derived from cross-sectional data. The authors appropriately acknowledge this limitation. Several points require further clarification.

1.1*) Many of the molecular features highlighted, including inflammatory and fibrogenic pathways and the proposed 57-feature panel, have been widely reported in MASLD. To better demonstrate the added value of this study, a clearer discussion of the novel biological insights and newly implicated genes from this analysis would strengthen the manuscript.*

R1.1 We thank the reviewer for this comment. We agree that several of the inflammatory and fibrogenic pathways highlighted in our study have already been linked to MASLD. Rather than weakening the study, we believe this is an important strength: because the analysis is fully data-driven, the independent recovery of these established disease-associated programmes supports the biological validity of the inferred trajectory.

The key advance of our work is not simply the re-identification of known MASLD markers, but the establishment of a continuous molecular disease trajectory that captures progression across the MASLD spectrum and enables patient positioning along this axis. This represents a conceptual shift from static stage-based classification toward a dynamic framework that better reflects the continuum of disease biology.

Within this framework, the 57-feature panel is intended as a minimal set of markers, detectable in plasma, that most effectively captures trajectory position, rather than as a catalogue of individually novel MASLD genes. Notably, however, the panel also contains several genes that have not been widely studied in MASLD, including NFASC, NCR3LG1, EIF3G, TPST1, AMY2B, and AOC1. These genes were selected based on their contribution to modelling disease progression and may therefore highlight underappreciated aspects of MASLD biology that warrant further investigation.

In response to the reviewer's suggestion, we have revised the Discussion to more clearly articulate the added value of the study, namely:

- the unbiased recovery of known MASLD-relevant programmes as validation of the model,
- the trajectory-based framework as the main conceptual innovation, and
- the emergence of less-characterised genes that may provide new biological hypotheses.

***1.2**) Inconsistency in Figure 2 requires clarification. Some modules described as positively associated with disease severity in Figure 2B appear to decrease along the pseudo-temporal axis in Figure 2C. The relationship between module–phenotype correlations and their trajectory patterns should be explained to help readers interpret these results.*

R1.2 We thank the reviewer for highlighting this point and agree that the distinction between these two panels was not sufficiently clear in the original version.

The values shown in Figure 2B are coefficients from multivariate linear models, not simple pairwise correlations between module expression and phenotype. These coefficients quantify the contribution of each module to the prediction of a given phenotypic feature while accounting for the other variables included in the model, including other modules, inflammation, and age. Consequently, the sign and magnitude of a coefficient reflect the conditional effect of that module within a multivariate framework and do not necessarily correspond to its overall expression trend along the pseudo-temporal axis.

By contrast, Figure 2C displays the observed expression dynamics of each module across the inferred trajectory, providing a direct view of how module activity changes over pseudo-time. Because these two panels address different questions, a module may show a positive coefficient in the multivariate model in Figure 2B while exhibiting a decreasing overall trend along the trajectory in Figure 2C.

To address this point, we have revised both the figure legend and the main text to explicitly clarify the distinction. The updated legend for Figure 2B now reads:

“Association (coefficient from a multivariate linear model) of the different gene co-expression modules with NAS score, steatosis, ballooning, and fibrosis. Red indicates a positive coefficient and blue a negative coefficient. These coefficients represent the contribution of each module to the model and should not be interpreted as the direction of module expression changes along the pseudo-temporal trajectory shown in panel C.”

We have also revised the corresponding Results section to improve interpretation.

***1.3)** While the authors attempt to distinguish cell-intrinsic regulatory changes from shifts in cellular composition, the current analyses do not fully address whether the inferred pseudo-temporal trajectory itself is primarily driven by changes in cell-type proportions. Although genes whose expression could be explained by cell composition were removed in downstream analyses, it remains unclear whether the trajectory (e.g., PC1 or the ordering framework) is largely defined by variation in immune infiltration or other compositional shifts. To strengthen the claim that the trajectory reflects true regulatory reprogramming rather than cellular proportion changes, the authors should explicitly assess the contribution of cell-type composition to the principal axis underlying the pseudo-temporal ordering. For example, examining correlations*

between the trajectory axis and cell-type fractions, performing variance partitioning, or reconstructing the trajectory after regressing out cell-type proportions would help clarify whether the observed progression is independent of compositional effects.

R1.3 We thank the reviewer for raising this important point. In bulk transcriptomic data it is difficult to fully disentangle cell-intrinsic regulatory changes from shifts in cellular composition. Importantly, our goal in constructing the trajectory was to capture the overall molecular progression of diseased liver tissue, which naturally reflects both regulatory changes within cells and alterations in cell-type composition that accompany disease progression.

In this context, changes in cellular composition are not merely a confounder but are themselves a consequence of underlying regulatory and pathological processes. Therefore, the trajectory was intentionally inferred from the global transcriptomic signal rather than attempting to remove compositional effects at this stage.

To better interpret the regulatory sources underlying the observed changes, we performed a cell-type deconvolution analysis to assess the extent to which gene expression patterns could be attributed to specific cellular populations and to highlight the biological processes involved. This analysis was intended to provide additional biological interpretation of the trajectory rather than to redefine or correct the trajectory itself.

We have clarified this point in the revised manuscript in the results section associated with defining the trajectory. "Because the trajectory was inferred from bulk liver transcriptomes, it reflects the overall molecular state of the tissue and therefore captures both cell-intrinsic regulatory changes and shifts in cellular composition that accompany disease progression."

Reviewer #2 (Remarks to the Author):

***2.1** The authors have addressed most of the previous concerns, and the innovative application of established computational methods has yielded numerous potentially interesting leads that may help deepen our understanding of disease progression mechanisms along the trajectory. However, the study appears better suited for hypothesis generation and motivating follow-up studies than for delivering clear actionable or translational insights at this stage.*

R2.1 We thank the reviewer for this thoughtful and balanced assessment. We agree that an important contribution of this study is to generate mechanistic hypotheses regarding MASLD progression and to provide a framework that can guide future functional and prospective studies. We also agree that the biomarkers identified here are not yet ready for immediate clinical application and will require prospective validation in appropriately designed cohorts.

At the same time, we believe the study offers value beyond mere hypothesis generation. First, the trajectory-based framework organises patients along a continuous molecular progression axis, enabling the identification of stage-specific regulatory programmes and pathways associated with different phases of disease progression. In this sense, the framework may help

prioritise biological processes and candidate targets that are most relevant at particular stages of MASLD evolution.

Second, the network-informed biomarker panel showed consistent performance across both transcriptomic and proteomic datasets and, in the datasets analysed here, outperformed commonly used clinical indices such as FIB-4. While this does not yet establish clinical utility, it supports the potential translational relevance of the approach and provides a rationale for future prospective validation.

To better reflect the reviewer's point, we have revised the Discussion to position the study as a framework that bridges mechanistic hypothesis generation and early translational prioritisation, while avoiding overstatement of its immediate clinical applicability. The revised text now states:

"Beyond generating mechanistic hypotheses, this framework enables the identification of stage-specific regulatory programs that may help prioritise candidate therapeutic targets and biomarkers for further investigation."

We believe this revised wording more accurately captures both the current scope of the study and its potential translational value.

***2.2** Several issues remain that require clarification or more cautious interpretation. In Figure 5B, the claim that the proposed model outperforms established clinical scores (FIB-4, APRI, and NFS) based on ROC-AUC is not supported by the data shown, as FIB-4 appears to perform better. This discrepancy should be clarified. Moreover, the proposed model demonstrates lower sensitivity than existing clinical tests. Given that sensitivity is often more clinically relevant than specificity for detecting advanced fibrosis, this limitation and its clinical implications should be discussed. Claims of "outperformance" should be supported by appropriate statistical evidence, including confidence intervals and formal tests comparing AUCs.*

R2.2 We thank the reviewer for pointing out this lack of clarity. In the Fujiwara dataset, the ROC–AUC of our model is comparable to that of FIB-4 and outperforms APRI and NFS. Proper statistical tests comparing AUCs now support the results. We agree that our text could give the impression that FIB-4 performs slightly better in this cohort. We have clarified this point in the revised manuscript and moderated the wording accordingly. "Specifically, in the Fujiwara cohort[35], the model achieved an AUC of 0.803 (0.794 for 15 genes), performing similarly or outperforming established clinical scores (FIB-4, APRI, NFS; **Fig. 5B**), while in the EPoS cohort[12] it reached an AUC of 0.86 (0.85 for 15 genes), exceeding the FIB-4 AUC by 10%...".

With respect to sensitivity and specificity, the reported values depend on the selected decision threshold. In the Fujiwara dataset, the threshold used for the biomarker panel results in slightly lower sensitivity but higher specificity compared with FIB-4. As the reviewer notes, sensitivity is an important consideration for detecting advanced fibrosis, and we have added text discussing this trade-off and its potential clinical implications.

The ROC–AUC values for the two approaches are very similar in the Fujiwara dataset (~0.80 for the proposed model vs ~0.81 for FIB-4; indeed the difference is not statistically significant according to the DeLong test), indicating comparable overall discriminatory performance. In the larger EPoS cohort, the biomarker panel shows improved performance across multiple metrics compared with FIB-4. We have clarified these points in the revised text and added confidence intervals for the ROC–AUC values, along with p-values from the nonparametric DeLong test comparing the ROC curves. Sensitivity, specificity, and accuracy are reported as point estimates at the selected threshold, consistent with common practice, and statistical comparisons are not applicable to these single-threshold metrics.

Finally, we would like to emphasise that the value of the biomarker panel extends beyond binary classification alone. In addition to classification performance, the panel enables positioning of patients along the inferred molecular disease trajectory, which may support more refined biological stratification beyond conventional fibrosis scores. We have nevertheless revised the manuscript to ensure that this added value is not presented as a substitute for rigorous comparison of clinical test performance.

***2.3** In Figure 4A, the interpretation that endothelial cell proportions remain relatively stable across the disease progression trajectory is not fully convincing. The figure instead suggests a clear upward trend, with endothelial cells increasing from approximately 15% to 30% at later stages. This pattern supports alternative biological interpretations, such as proliferation of liver sinusoidal endothelial cells or expansion of dysfunctional endothelial populations, which warrant discussion.*

R2.3 We thank the reviewer for raising this point. Based on our interpretation, Figure 4A does not show a large increase in endothelial cell proportions across the trajectory. Rather, endothelial cells remain relatively stable, increasing only slightly from approximately 6% to 6.8% of the total inferred cellular composition (Figure included inline with this response for convenience). A similar pattern is observed in Supplementary Figure 8.

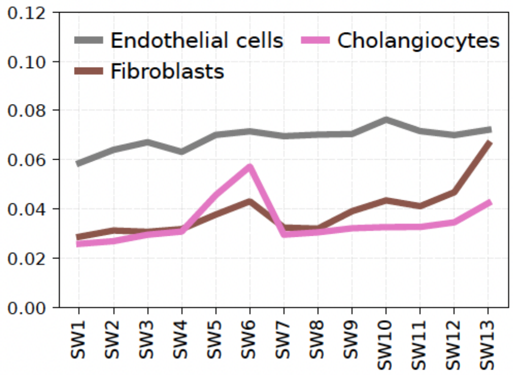

***2.4** Finally, the use of the term "genetic evidence" is not sufficiently justified. Establishing genetic evidence for causality generally requires analyses such as colocalization and Mendelian randomization using appropriate instrumental variables. Mapping to GWAS signals alone is insufficient to support causal claims and should therefore be described more cautiously.*

R2.4 We thank the reviewer for this important clarification. We agree that establishing genetic evidence for causality typically requires analyses such as colocalization or Mendelian randomization using appropriate instrumental variables, which were beyond the scope of this study. In the present study, our intention was to highlight overlap between genes identified

through the trajectory-based analysis and loci previously implicated in MASLD-related GWAS, rather than to claim causal genetic evidence.

To avoid overinterpretation, we have revised the manuscript to replace the term “genetic evidence” with more cautious wording (e.g., “genetic support” / “overlap with GWAS loci”) and clarified that these results indicate consistency with existing genetic studies rather than causal inference: ”Although GWAS overlap alone does not establish causality, the enrichment of trajectory-associated genes near MASLD GWAS loci supports consistency with existing genetic studies”. Furthermore, in the Discussion, instead of claiming genetic support, we wrote: “linking circulating markers to disease-associated regulatory programmes and to genes supported by MASLD GWAS associations”.

Reviewer #2 (Remarks on code availability):

The authors have added the code I previously requested. I have not tried to run the code myself.

Reviewer #3 (Remarks to the Author):

The article "Decoding MASLD Progression: A Molecular Trajectory-Based Framework for Modelling Disease Dynamics" by Kamzolas et al applies a biostatistical modeling approach to categorizing stages of disease in MASLD. The authors employ a number of techniques using available transcriptomic, proteomic, phenotypic, and histologic datasets to ascertain a molecular disease signature across the MASLD continuum. While the methodology, hypothesis, and clinical applications are of interest to the general reader and to liver disease specialists, the article lacks basic cellular biology techniques that align with the journal. Minor comments include a need to reduce the number of supplemental figures and cohere the critical pieces of the data.

Minor comments:

***3.1** There is a need to reduce the number of supplemental figures and cohere the critical pieces of the data.*

R3.1 We thank the reviewer for the suggestion. At the editor’s request we have turned the supplementary figures into Extended figures and only kept one Supplementary Figure.

***3.2** It is unclear why the authors chose the specific datasets they did (there are numerous available... what was the rationale for use of the ones chosen to build this model?)*

R3.2 We thank the reviewer for this question. The datasets included in this study were selected based on a combination of scientific relevance and practical suitability for trajectory modelling and validation. Specifically, we prioritised cohorts that met the following criteria:

(i) the availability of liver transcriptomic data with accompanying histological annotations (NAS score, steatosis, ballooning, and fibrosis stage),
(ii) sufficient sample size and representation across different stages of MASLD, and
(iii) suitability for independent validation analyses.

Our aim was not to exhaustively include all available datasets, but to use a set of cohorts that provided the most appropriate combination of histological depth, disease-stage coverage, and cross-cohort comparability for the specific questions addressed here. The selected cohorts allowed us to construct the trajectory framework in one dataset and evaluate its robustness across independent cohorts with differing patient characteristics.

We have clarified the rationale for dataset selection in the revised Methods section.”The above-described datasets were selected based on the availability of liver transcriptomic data with detailed histological annotation and sufficient coverage across MASLD stages, allowing both model development and independent validation.”

3.3 *It is unclear why the authors chose the putative signature genes a priori (for eg, what is the rationale for choosing the candidate biomarkers in Fig 5A?)*

R3.3 We thank the reviewer for pointing out that this step was not sufficiently explained. Our aim was to identify candidate biomarkers that are both mechanistically linked to MASLD progression and detectable in circulation.

These genes were not selected a priori based on prior literature or manual curation. Rather, they were prioritised through a stepwise strategy designed to identify markers that are both biologically linked to MASLD progression and potentially measurable in circulation.

Specifically, we prioritised genes that satisfied two criteria. First, they had to be part of the MASLD regulatory network inferred in this study, ensuring that the selected markers were connected to disease-associated regulatory programs rather than representing arbitrary predictive features. Second, we required that the corresponding proteins show correlated abundance between liver transcriptomic expression and plasma proteomic measurements, as reported in the dataset of Govaere et al., thereby increasing the likelihood that the markers would be detectable in plasma and suitable for non-invasive monitoring.

The candidate biomarker set used in Fig. 5A therefore represents the intersection between genes present in the MASLD regulatory network and genes whose transcript abundance correlates with circulating protein levels. We have clarified this rationale in the revised manuscript by rewriting the second paragraph of the relevant results section.

3.4 *How relevant is it that this gene-based biomarker that applies across the MASLD continuum outperforms FIB-4, which was only established to predict fibrosis?*

R3.4 We thank the reviewer for this important point. We agree that FIB-4 was originally developed to predict advanced fibrosis, whereas the biomarker panel proposed here aims to capture molecular changes across the MASLD continuum. The comparison with FIB-4 was therefore not intended to suggest identical clinical use cases, but rather to benchmark the performance of the biomarker panel against a widely used non-invasive clinical score.

Importantly, beyond fibrosis prediction, the biomarker panel provides additional information by positioning patients along the inferred molecular disease trajectory, which may allow a more refined characterisation of disease state across the MASLD spectrum. We have clarified this distinction in the revised manuscript: "Although FIB-4 was originally developed for the detection of advanced fibrosis, we include it here as a commonly used non-invasive clinical benchmark."

3.5 *Were there any novel pathways found besides inflammation and fibrosis? Both of whose signatures increase along the histologic disease continuum?*

R3.5 We thank the reviewer for this question. In addition to inflammatory and fibrogenic processes, our trajectory analysis identified a broad range of pathways associated with MASLD progression (Supplementary Table 13). These include pathways related to metabolic regulation (e.g., integration of energy metabolism, mitochondrial biogenesis, heme signaling), cellular stress responses (e.g., unfolded protein response and protein folding), epigenetic regulation (chromatin modifying enzymes and gene silencing by RNA), intracellular trafficking (membrane trafficking and Golgi–ER transport), and developmental signaling pathways such as WNT, NOTCH, and Hedgehog.
Several of these processes have been less systematically characterised in MASLD progression studies. For example, we found processes related to proteostasis (unfolded protein response), mitochondrial regulation (mitochondrial biogenesis, peroxisomal protein import), epigenetic control (chromatin-modifying enzymes), and intracellular trafficking. While these pathways have been implicated in MASLD, our framework reveals how they are dynamically regulated along the disease trajectory, rather than only at isolated histological stages. These results have been outlined in the respective results section (line 305 onwards).

Importantly, the primary contribution of our study is not the identification of individual pathways per se, but the reconstruction of a continuous molecular trajectory of MASLD progression. This framework allows regulatory programs—including inflammatory, fibrotic, metabolic, and signaling pathways—to be placed within a unified progression model and to be linked to stage-specific regulatory networks and circulating biomarkers.

Reviewer #4 (Remarks to the Author):

I thank the authors for addressing my comments. The manuscript reads well and is defensible.

4.1 *My only final question/comment is that the shift from histological modeling of MASLD progression to transcriptomic modeling seems like a major advance. This is not my area of*

expertise, so I did some quick searches to see if these ideas have been explored before. These papers seem to at minimum lay the groundwork for the current study:

Characterization of transcriptional modules related to fibrosing-NAFLD progression
Yi Lou, Guo-Yan Tian, Yu Song, Yin-Lan Liu, Yi-Dan Chen, Jun-Ping Shi & Jin Yang

Development of a novel non-invasive biomarker panel for hepatic fibrosis in MASLD
Lars Verschuren et al

Hepatic gene expression profiles differentiate presymptomatic patients with mild versus severe nonalcoholic fatty liver disease
Cynthia A Moylan et al

Human hepatic gene expression signature of non-alcoholic fatty liver disease progression, a meta-analysis
Maria Ryaboshapkina & Mårten Hammar

Pseudotime Analysis Imputes the Missing Liver NAFLD Status in Public RNA-Seq Cohorts
Tongyang Wang, Xiangmei Dou

I couldn't find citations for these in the manuscript. My request is whether these (and other cited studies that have done transcriptomic analysis and/or temporal analysis of MASLD) could be cited in the introduction or conclusion, to more clearly contextualize what the current study adds on top of them. Or, if these studies are not relevant, that would be fine to address as well.

R4.1 We thank the reviewer for highlighting these relevant studies and apologise for omitting several of these citations in the original manuscript. Several previous works have used transcriptomic analyses to investigate molecular signatures associated with MASLD severity or progression, including studies identifying transcriptional modules linked to fibrosing NAFLD progression, gene expression differences between disease stages, and meta-analyses of transcriptomic datasets. We agree that these studies provide important context for the field and have now cited them in the revised Introduction.

While these studies have provided valuable insights into transcriptional changes associated with MASLD, most focused on identifying differential expression signatures or gene modules associated with predefined histological stages, rather than reconstructing disease progression as a continuous molecular trajectory. In addition, these approaches generally did not integrate regulatory network inference or connect transcriptomic changes with circulating biomarkers. To our knowledge, this is the first study to integrate pseudotemporal ordering, regulatory network inference, and cross-omics biomarker mapping into a unified framework for MASLD progression.

We have also cited the study applying pseudotime analysis to NAFLD datasets. That work demonstrated the feasibility of applying trajectory-based approaches to bulk liver transcriptomic data but was primarily aimed at imputing disease status in heterogeneous cohorts. In contrast, the goal of our study was to reconstruct a reference molecular trajectory of MASLD progression,

integrate this trajectory with regulatory network analysis, and use this framework to identify stage-specific regulatory programs and candidate circulating biomarkers across multiple cohorts. This trajectory-based framework therefore extends previous transcriptomic studies by linking molecular progression with regulatory network context and non-invasive biomarker discovery.

Finally, regarding the study by Verschuren et al., we agree that this work is directly relevant and should have been considered in our analysis. We apologise for the omission and have now evaluated the performance of the three biomarkers proposed in that study alongside our biomarker panel. The results of this comparison have been added to the revised Figure 5, showing that our biomarker panel outperforms this 3-gene panel (results supported by statistical analysis using the DeLong test comparing the AUCs under the respective ROC curves).
